# The Localization Behavior of Different CNTs in PC/SAN Blends Containing a Reactive Component

**DOI:** 10.3390/molecules26051312

**Published:** 2021-03-01

**Authors:** Marén Gültner, Regine Boldt, Petr Formanek, Dieter Fischer, Frank Simon, Petra Pötschke

**Affiliations:** Leibniz-Institut für Polymerforschung Dresden e.V. (IPF Dresden), Hohe Str. 6, 01069 Dresden, Germany; Maren.Gueltner@stfi.de (M.G.); boldt@ipfdd.de (R.B.); formanek@ipfdd.de (P.F.); fisch@ipfdd.de (D.F.); frsimon@ipfdd.de (F.S.)

**Keywords:** nanocomposites, carbon nanotubes, polymer blends, melt mixing, polycarbonate, poly(styrene-*co*-acrylonitrile), reactive additive, carbon nanotube localization, morphology

## Abstract

Co-continuous blend systems of polycarbonate (PC), poly(styrene-*co*-acrylonitrile) (SAN), commercial non-functionalized multi-walled carbon nanotubes (MWCNTs) or various types of commercial and laboratory functionalized single-walled carbon nanotubes (SWCNTs), and a reactive component (RC, *N-*phenylmaleimide styrene maleic anhydride copolymer) were melt compounded in one step in a microcompounder. The blend system is immiscible, while the RC is miscible with SAN and contains maleic anhydride groups that have the potential to reactively couple with functional groups on the surface of the nanotubes. The influence of the RC on the localization of MWCNTs and SWCNTs (0.5 wt.%) was investigated by transmission electron microscopy (TEM) and energy-filtered TEM. In PC/SAN blends without RC, MWCNTs are localized in the PC component. In contrast, in PC/SAN-RC, the MWCNTs localize in the SAN-RC component, depending on the RC concentration. By adjusting the MWCNT/RC ratio, the localization of the MWCNTs can be tuned. The SWCNTs behave differently compared to the MWCNTs in PC/SAN-RC blends and their localization occurs either only in the PC or in both blend components, depending on the type of the SWCNTs. CNT defect concentration and surface functionalities seem to be responsible for the localization differences.

## 1. Introduction

As new applications for polymer-based materials emerge, the demands on their performance also increase. Both the blending of different commercial polymer types and the production of nanocomposites are suitable ways to meet these requirements. In polymer blends, the typical properties of the parent polymers can be combined and synergistic effects may be exploited. Thereby, in immiscible polymer blends, which comprise the majority of blend systems, the blend morphology type plays an important role on the property profile [1,2]. In this context, especially the co-continuous blend morphology is often advantageous. If such morphology is obtained, the properties of both polymers can be combined synergistically as each polymer forms a continuous matrix [3]. For example, property maxima were obtained for impact strength at blend compositions resulting in co-continuous morphology [4], and higher tensile moduli than in dispersed structures [5,6].

The incorporation of fillers into polymers can help to increase mechanical performance and stability and reduce the price [7]. In the case of layered fillers, barrier properties may also be incorporated, e.g., in films [8]. In recent years, nanofillers with new functionalities have come into particular focus. For example, carbon-based nanofillers can introduce the property of electrical and thermal conductivity into typically insulating polymers [9,10]. Furthermore, mechanical strengthening is possible if suitable interfacial adhesion is achieved in addition to good dispersion. Due to their high aspect ratio and intrinsic electrical, mechanical, and thermal properties, carbon nanotubes (CNTs) have recently been widely used to modify polymer matrices. Such an addition can favorably influence not only the electrical and thermal properties, but also the mechanical properties, scratch and wear behavior, oxidation stability, and flame retardancy [9,11,12,13].

Combining both approaches, blending and the addition of nanofillers like multi-walled CNTs (MWCNTs), can result in additional property effects. This is especially the case when electrical conductivity of the blend matrix is desired. With blends having a co-continuous morphology, typically a lower filler concentration is needed compared to a single composite. This is due to the fact that nanotubes tend to localize in the better wetting component of the blend. If this component is continuous, and the filler concentration in this component reaches the electrical percolation threshold, the whole blend composite is electrically conductive. This concept of double percolation was first introduced by Sumita et al. [14] using immiscible polymer blends with carbon black (CB). This concept was later used in systems containing other conductive fillers, such as CNTs [15,16,17,18,19]. This selective localization behavior of nanotubes in polymer blends in general is most commonly explained by thermodynamic reasons and the different interfacial interactions between the matrix components with the nanotubes. To estimate the possible filler localization, the concept of the wetting coefficient is often applied [14,20,21,22,23,24,25,26,27,28,29], with better or worse correlation with practical results. However, besides thermodynamic preferences, kinetic aspects may also play a role on the final filler localization, especially in non-equilibrium systems, such as melt-mixed composites [30,31,32]. If the filler is pre-localized in the thermodynamically less preferred component, or localizes there due to a lower melting or softening temperature of this component, a too short mixing time and too high melt viscosity may hinder reaching the thermodynamically favorable localization [32,33,34]. By adapting the mixing sequence and conditions in a suitable way, it is even possibly to localize such nanofillers at the interface [26,31,33,35,36,37,38,39]. Kinetic hindrance of migration from a non-preferred to a preferred component can also occur, as in the case of spherical particles, such as carbon black and nanotube agglomerates or layered structures, such as phyllosilicates and nano-graphite, resulting in interface localization [40,41,42].

In order to enhance the mechanical properties of immiscible blends, quite often compatibilizers, especially reactive ones, are added. They are expected to refine the blend morphology and to enhance interfacial adhesion. If used in nanocomposite blends, depending on their miscibility with the blend components and localization behavior, they also can help to improve the nanofiller dispersion and can affect the localization behavior. A first example of localizing nanotubes at the blend interface by premixing with a reactive compatibilizer was shown by Bose et al. in polyamide 6 (PA6)/acrylonitrile- butadiene-styrene (ABS) blends [43]. In this case, the common reactive compatibilizer styrene maleic anhydride copolymer (SMA) was applied. Maleic anhydride (MA) groups not only improve the polarity of non-polar polymers, such as polyolefins, but also can interact and react with polymer matrices and nanotubes containing functional groups, such as amino end groups in polyamides or functional groups on nanotube surfaces. For example, the properties of polypropylene (PP)/MWCNT composites could be enhanced by using MA grafted polypropylene (PP-*g*-MA) by strong hydrogen bonding between hydroxyl groups of the MWCNTs and MA groups of PP-*g*-MA [44,45]. Another example for enhancing adhesion of CNTs to the polymer matrix was shown by Wang et al. who used SMA grafted MWCNTs in poly(vinyl chloride) (PVC) [46].

For the blend system polycarbonate (PC)/poly(styrene-*co*-acrylonitrile) (SAN) under consideration in this paper, Göldel et al. [20] described double percolation at the PC/SAN composition of 40/60 wt.% and added MWCNT Baytubes^®^ C150HP. The MWCNTs showed selective localization in the PC component, regardless of the component in which the CNTs were first incorporated. This localization was explained as thermodynamically induced, as the interfacial interactions between MWCNTs and PC are stronger than those between MWCNTs and SAN. Even if the MWCNTs were premixed in the thermodynamically less preferred SAN component, they migrated rapidly during the melt-mixing into the PC component by crossing the interface [34], whereas spherical carbon black particles tend to stack at the blend interface [40] which was explained by the “slim-fast mechanism”.

With the aim to tune the CNT localization in the PC/SAN blend system and to achieve CNT localization in the SAN component, Gültner et al. [47] added a reactive component (*N*-phenylmaleimide styrene maleic anhydride copolymer, RC), which is miscible with the SAN and studied the localization of amino-functionalized MWCNTs Nanocyl™NC3152 after the melt-mixing. It was expected that the MA group in the RC, even if diluted in the SAN, can fix the nanotubes in this component due to interactions or even reactions between the amino groups of the CNTs with the MA functionality. In blends without this RC, this kind of CNTs also was localized in the PC component of the SAN/PC blend. The nanotube localization in the SAN-RC/PC blend systems at different weight ratios of RC and MWCNTs showed that selective localization in the SAN-RC component could be obtained above a certain critical ratio between amino functionalized MWCNTs and RC. They also found that the CNT localization behavior is dependent on the mixing sequence at low RC/CNT ratios, which indicates a chemical coupling or strong interactions between CNTs and RC as the reason for this localization behavior.

To follow up on this exciting result and the opportunity of tuning MWCNT localization by simple addition of a reactive component, it is of interest to study how non-functionalized MWCNTs and single-walled CNTs (SWCNTs) behave concerning their localization behavior. With this purpose, in this study, PC_60_/SAN_40-x_-RC_x_-blends were prepared by one-step melt-mixing using the following MWCNT materials: non-functionalized MWCNTs Nanocyl™ NC7000 and NC3150 (in comparison to NC3152), non-functionalized MWCNTs of the type Baytubes^®^ C150P and C150HP, and two differently graphitized NC7000 materials, NCg-7000-1 and NCg-7000-2. In addition, different SWCNT materials were applied, HiPCo^TM^, SWeNT^®^ CG100, AP-SWNT, and amino-functionalized AP-SWNT-NH_2_. All blend composites contained 0.5 wt.% of MWCNT or SWCNT. Transmission electron microscopy (TEM) was used to determine the localization. Furthermore, possible reasons for the different localization behavior were studied using a characterization of the CNT materials using IR and Raman spectroscopy and X-ray photoelectron spectroscopy (XPS) analysis; additional effects caused by the blend partners are discussed.

## 2. Results

### 2.1. Localization Behavior of Nanocyl™ MWCNTs

**Figure 1:**
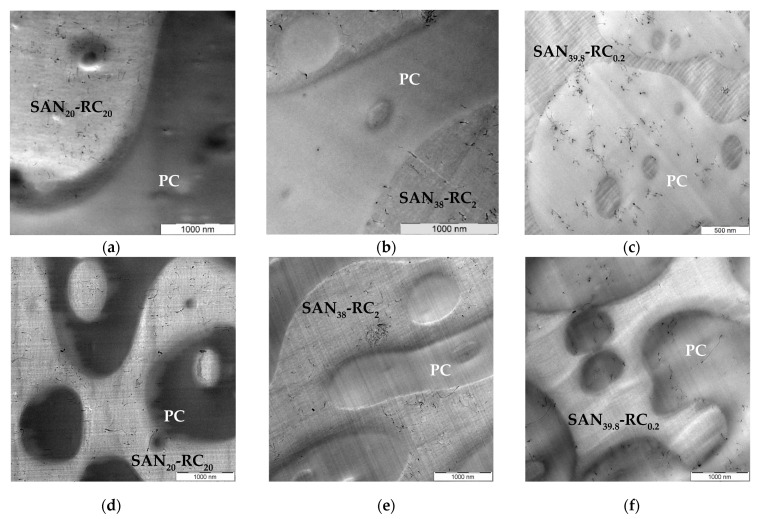
TEM images of PC_60_/SAN_40_-x-RCx blends filled with NC3150 (**a**–**c**) and NC7000 (**d**–**f**): (**a**) PC_60_/SAN_20_-RC_20_ blends and (**b**) PC_60_/SAN_38_-RC_2_ blends illustrating NC3150 (0.5 wt.%) in SAN-RC; and (**c**) in PC_60_/SAN_39.8_-RC_0.2_ blends NC3150 (0.5 wt.%) stay in PC; (**d**) PC_60_/SAN_20_-RC_20_ blends and (**e**) PC_60_/SAN_38_-RC_2_ blends showing NC7000 (0.5 wt.%) in SAN-RC; and (**f**) in PC_60_/SAN_39.8_-RC_0.2_ blends NC7000 (0.5 wt.%) stay in PC.

In the previous study [47], the influence of increasing amounts of the reactive component (RC) on the localization of amino-functionalized CNTs Nanocyl™ NC3152 in PC/SAN-RC (60/40) blends was studied. For comparison, different kinds of MWCNTs from Nanocyl™, the as-produced non-functionalized MWCNT NC7000 and the therefrom prepared purified and shortened, but still non-modified MWCNT NC3150 types were applied. NC3150 is the non-functionalized counterpart to amino functionalized NC3152. In PC_60_/SAN_40_, NC3150 localizes in PC. In Figure 1, the localization behavior of NC3150 in the PC_60_/SAN_40_-x-RC_x_ blend with the variation of the RC amount is illustrated. The assignment of the components is based on the roughness of the thin sections for TEM. The PC part is smooth, whereas the more brittle SAN-RC part is ruffled [20]. This assignment is further confirmed by energy filtered TEM (EF-TEM, as in Figures S1–S3). The MWCNTs are located in SAN-RC after the addition of 20 wt.% and 2 wt.% RC (Figure 1a,b). However, when introducing only 0.2 wt.% RC, CNT localization in PC was observed (Figure 1c) and only a few CNTs in small agglomerates were found in SAN-RC. Unexpectedly, this is the same localization behavior as for the previously studied amino-functionalized MWCNTs Nanocyl™ NC3152 [47]: CNT localization in SAN-RC when 20 wt.% or 2 wt.% RC was added and CNT localization in PC when only 0.2 wt.% RC or no RC was added.

When using the as-produced NC7000, nominally also non-functionalized, they are also localized in the SAN-RC component of the PC_60_/SAN_20_-RC_20_ blend (Figure 1d) and the PC_60_/SAN_38_-RC_2_ blend (Figure 1e). In contrast to that, again, 0.2 wt.% RC addition resulted in localization in PC (Figure 1f). Thus, the localization behavior of these non-functionalized MWCNTs also depends on the RC concentration.

Based on the hypothesis that functional groups existent on the MWCNT surface may be responsible for this localization behavior, two types of MWCNT were modified from the same starting product, namely NC7000. The nanotube material was graphitized in an oven at 2600 °C under argon atmosphere for 1 hour, as recommended by Zeng et al. [48], and named NCg-7000-1. Based on the reference it was expected that all oxygen containing groups on the surface of the MWCNTs are eliminated by this step. However, in order to enhance the effectivity of the annealing step, the graphitized material NCg-7000-1 was treated second time under the same conditions (2600 °C and argon atmosphere); the product was named NCg-7000-2.

**Figure 2:**
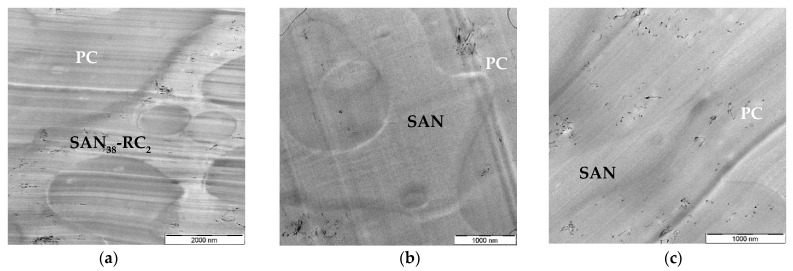
TEM images of PC_60_/SAN_40_-_x_-RC_x_ blends filled with NCg-7000-1 (**a**,**b**) and NCg-7000-2 (**c**): (**a**) PC_60_/SAN_38_-RC_2_ blends showing NCg-7000-1 (0.5 wt.%) in SAN-RC; and (**b**) in PC_60_/SAN_40_ blends NCg-7000-1 and (**c**) in PC_60_/SAN_40_ blends NCg-7000-2 (0.5 wt.%) stay in PC.

Then, the graphitized MWCNTs NC7000 were applied and their localization behavior was studied. The TEM investigations (Figure 2 and Figure S1) indicate that both types localized selectively in one of the blend components, again depending on whether RC is added or not. For this example, the component assignment was proven by EF-TEM analysis as shown in Figure S1 (In the Supplementary Material). Without RC, both types of graphitized NC7000 localize in PC (Figure 2b,c) and with the addition of 2 wt.% RC localization in SAN-RC was achieved (Figure 2a) as well as with 20 wt.% RC (Figure S1).

### 2.2. Localization Behavior of Baytubes^®^ MWCNTs

Furthermore, to complete this investigation and show that this localization behavior is not only an effect of Nanocyl™ MWCNT products, the localization behavior of Baytubes^®^ MWCNTs was studied. For this, Baytubes^®^ filled PC/SAN-RC blends were prepared varying the RC content, to get blends in the ratios of 60/20-20 wt.%, 60/38-2 wt.% or 60/39.8-0.2 wt.%.

**Figure 3:**
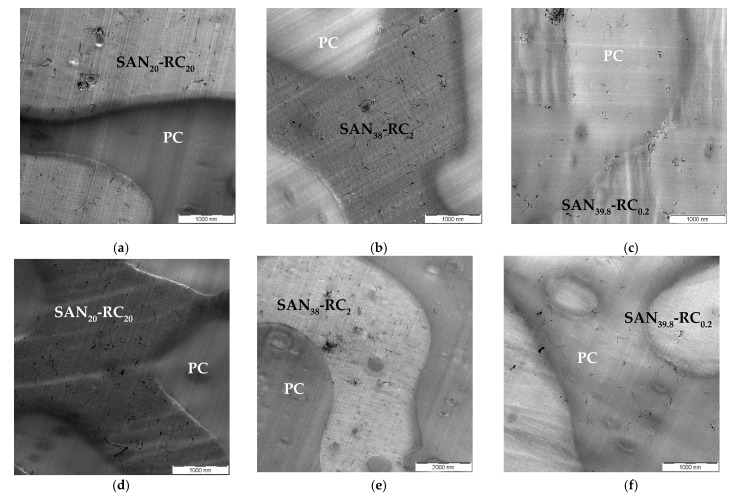
TEM images of PC_60_/SAN_40-x_-RC_x_ blends filled with Baytubes^®^ C150P (**a**–**c**) and C150HP (**d**–**f**): (**a**) PC_60_/SAN_20_-RC_20_ blends and (**b**) PC_60_/SAN_38_-RC_2_ blends showing Baytubes^®^ C150P (0.5 wt.%) localized in SAN-RC, (**c**) in PC_60_/SAN_39.8_-RC_0.2_ blends Baytubes^®^ C150P (0.5 wt.%) stay in PC, (**d**) PC_60_/SAN_20_-RC_20_ blends and (**e**) PC_60_/SAN_38_-RC_2_ blends showing Baytubes^®^ C150HP (0.5 wt.%) localized in SAN-RC, (**f**) in PC_60_/SAN_39.8_-RC_0.2_ blends Baytubes^®^ C150HP (0.5 wt.%) stay in PC.

The TEM investigations (Figure 3) show that, in PC_60_/SAN_20_-RC_20_ (Figure 3a,d) and PC_60_/SAN_38_-RC_2_ (Figure 3b,e), the 0.5 wt.% Baytubes^®^ of both types (C150P and C150HP) localize in SAN-RC. In the PC_60_/SAN_39.8_-RC_0.2_, the localization behavior is reversed; most of the Baytubes^®^ stay in PC, only few MWCNTs are visible at the interface or in SAN-RC (Figure 3c,f). In contrast to the Nanocyl™ NC7000, there are more small agglomerates in SAN-RC and more MWCNTs at the interface.

### 2.3. Localization Behavior of SWCNTs

**Figure 4:**
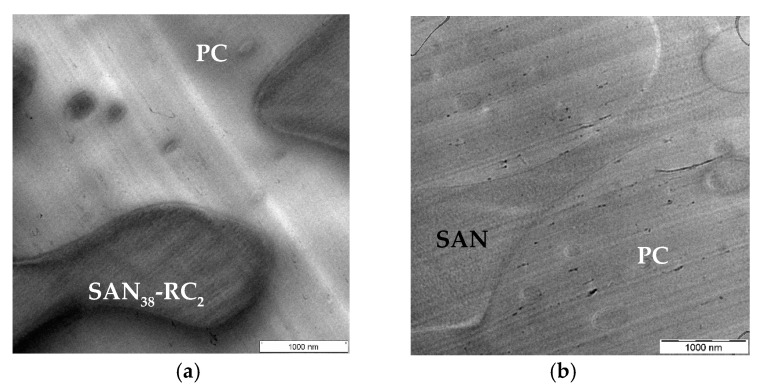
TEM images of HiPCo^TM^ SWCNT filled PC_60_/SAN_40-x_-RC_x_ blends: (**a**) PC_60_/SAN_38_-RC_2_ blends showing HiPCo^TM^ SWCNTs (0.5 wt.%) localized in PC and (**b**) in PC_60_/SAN_40_ blends HiPCo^TM^ SWCNTs (0.5 wt.%) stay in PC.

**Figure 5:**
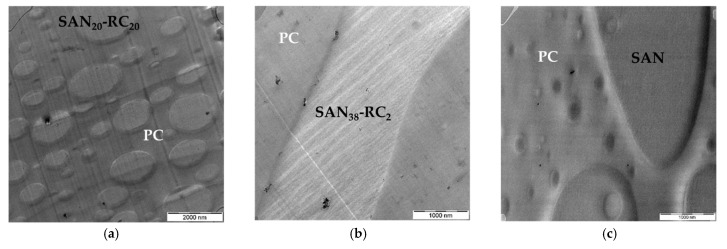
TEM images of SWeNT^®^ SWCNT filled PC_60_/SAN_40-x_-RC_x_ blends: (**a**) PC_60_/SAN_20_-RC_20_ blends and (**b**) PC_60_/SAN_38_-RC_2_ blends showing SWeNT^®^ (0.5 wt.%) localized in both components and (**c**) in PC_60_/SAN_40_ blends SWeNT^®^ (0.5 wt.%) remain in PC.

**Figure 6:**
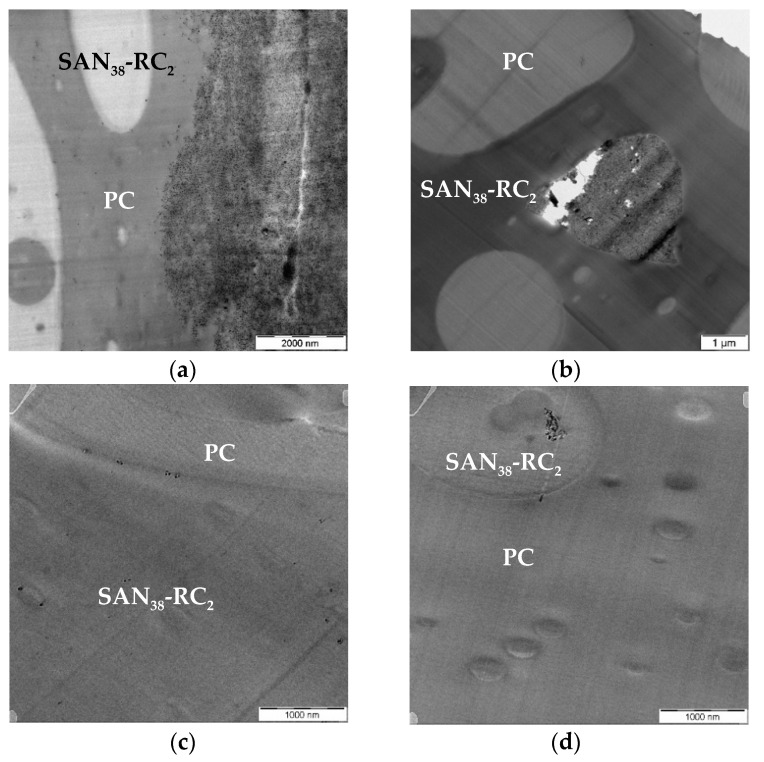
TEM images of PC_60_/SAN_38_-RC_2_ blends filled with AP-SWNT and AP-SWNT-NH_2_: (**a**,**c**) PC_60_/SAN_38_-RC_2_ blends showing AP-SWNT (0.5 wt.%) localized in both components with most of the SWCNTs in PC, (**b**,**d**) in PC_60_/SAN_38_-RC_2_ blends AP-SWNT-NH_2_ (0.5 wt.%) localized in SAN-RC.

Additionally, in order to check if there are any differences between SWCNTs and MWCNTs, the localization behavior was studied for different kinds of SWCNTs (Figure 4, Figure 5 and Figure 6). At first, high-purity HiPCo^TM^ SWCNTs were incorporated in PC_60_/SAN_40_-_x_-RC_x_ blend. For this purpose, a blend with the 2 wt.% RC was used to study the SWCNT localization behavior. This RC content is higher than the critical RC ratio where the CNT localization inverted in the case of all investigated MWCNTs. In contrast to these, the TEM observations as shown in Figure 4 reveal SWCNT localization in PC (Figure 4a), the same localization as in non-modified PC_60_/SAN_40_ blends (Figure 4b). In general, the visibility of the SWCNTs is much worse than that of MWCNTs due to the much thinner diameter of dispersed SWCNTs. However, larger agglomerates were also found (see, e.g., those shown for AP-SWNTs in Figure 6) which typically are not within a TEM cut, but reduce the number of nanotubes available for dispersion. As reason for this different localization behavior of HiPCo^TM^ as compared to MWCNTs, it is assumed that the high-purity HiPCo^TM^ SWCNTs may not have functional groups at the surface which could couple towards the maleic anhydride groups of RC as it was the case for all investigated MWCNTs. This is studied and discussed later.

For the next study, SWCNTs SWeNT^®^ CG100 were incorporated in the PC_60_/SAN_40-x_-RC_x_ blends. Surprisingly, even if most of these SWCNTs localized in PC, a few of them localized in SAN_38_-RC_2_ or at the interface (Figure 5b). It may be that the concentration of RC is too low for the much higher number of SWCNTs compared to the same amount of MWCNTs. Therefore, a higher concentration of RC in SAN, namely the PC_60_/SAN_20_-RC_20_ blend, was also investigated. The TEM image (Figure 5a) shows that the SWeNT^®^ are also at this high RC content localized in both blend components. This implies that for this type of SWCNTs the localization does not depend on the ratio between the concentration (or number) of SWCNTs and the RC (within the studied range). However, in the non-modified PC_60_/SAN_40_ blend the SWeNT^®^ localized, like all other CNTs studied, selectively in PC (Figure 5c).

To gain a deeper understanding for this different localization behavior, a third kind of SWCNTs with the designation AP-SWNT and therefrom prepared amino functionalized SWCNT AP-SWNT-NH_2_ were applied. The AP-SWNT material was produced by an electric arc process and the producer promotes this product as the material, which has the “highest purity of any commercially available AP-EA-SWNT” [49] (here, AP stands for as produced, and EA for electric arc). The TEM results of the corresponding PC_60_/SAN_38_-RC_2_ blends with 0.5 wt.% AP-SWNT and AP-SWNT-NH_2_ are shown in Figure 6 and Figure S2 (In the Supplementary Material) and indicate a significant amount of undispersed nanotubes, seen as agglomerate areas in both samples. The larger and smaller SWCNT agglomerates of AP-SWNT seem to preferentially localize in PC. However, some of the individual nanotubes appear to have the same localization behavior like the SWeNT^®^ SWCNT material, namely in both components or near the interface (Figure 6a,c). For the NH_2_ modified SWCNT material, individualized tubes are localized in SAN-RC (Figure 6b,d, Figure S3 (in the Supplementary Material). For these blends, the component assignment was more difficult; thus, EF-TEM was used for clear identification as shown in Figures S2 and S3, in the Supplementary Material.

A summary of the obtained localization of 0.5 wt.% of the different CNTs in blends with different RC contents is given in Table 1.

## 3. Discussion

### 3.1. Reasons for Localization Changes

**Figure 7:**
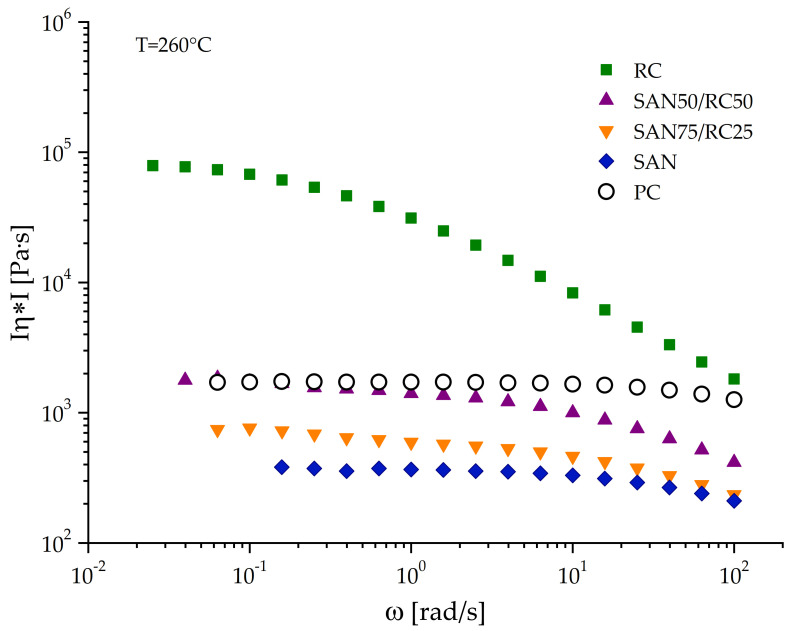
Complex melt viscosity |η*| in dependence of oscillation frequency at 260 °C for the different blend components.

Localization changes of different nanotube types were found after adding a reactive component to co-continuous PC/SAN blends. Whereas in blends without the RC all nanotube types stayed in PC, after RC addition CNTs were also found in SAN-RC or at the interface, depending on RC amount and CNT type. For such changes of the localization behavior, different reasons may be responsible. The addition of RC to SAN changes its melt viscosity at the processing temperature, as RC has a higher viscosity than SAN. The complex viscosity curves of SAN, RC and different mixtures at the processing temperature of the blends are shown in Figure 7.

In PC/SAN blends, PC is the polymer with the higher melt viscosity. Even though the literature describes a preference for localizing carbon fillers in the lower viscosity component [51], in this case, SAN, which also softens first, all nanotube types in unmodified blends localize in the higher viscosity PC for thermodynamic reasons. Addition of RC enhances the melt viscosity of the SAN/RC mixture; however, the melt viscosity does not get higher than that of PC, especially at higher frequencies relevant for the mixing process. Thus, the viscosity ratio between PC and SAN/RC stays lower than one, even if the viscosities are approaching with increasing RC content. This implies that the changes in melt viscosity upon RC addition cannot be the reason for the localization change.

A second reason can be that the surface energy of SAN is changing when adding RC. The surface energy of the blend components and of the nanotubes determine the CNT localization in equilibrium. In PC/SAN blends, calculations of the wetting coefficients showed that there is a clear tendency of the nanotubes to localize in PC, even when taking different CNT surface energy values from literature and different calculating equations [20]. In order to investigate such possible changes, the surface energies of the here investigated polymer materials were studied using contact angle measurements of different liquids on compression molded polymer samples [52]. From those values, surface energies and their polar and distributive parts were calculated according to Owens and Wendt. These values were used to calculate interfacial energies between the polymer pairs and between the polymers and CNTs, using both the harmonic and geometric mean methods. The measured surface energies are shown in Table S2, the calculated interfacial energies between the polymer pairs in Table S3. According to these measurements, SAN has the higher surface energy and slightly higher polarity than PC. Adding RC reduces the surface energy of SAN whereas the polarity stays nearly constant. At 0.5 wt.% RC addition to SAN, the surface energy of SAN-RC is the same as that of PC. At higher RC contents the values of the mixtures are below that of PC. Therefore, the interfacial tension between PC and SAN-RC decreases to a very low values when 0.5 wt.% RC is added and increases again with further increase in RC content. The calculation of the interfacial energies between the blend components and the nanotubes is not possible, as surface energy values for the applied CNTs are not available. They are expected to be different from values given in the literature and depending on the kind and functionalization of the nanotubes. Two most commonly used data sets from literature for non-modified nanotubes are given by Barber et al. [53] and Nuriel et al. [54]. Application of these data results in quite different values for the interfacial tensions between both polymer blend parents and the nanotubes. The calculation of wetting coefficients is only in general accordance with the observations for the values provided by Barber et al. [53], having lower summary and polar contribution of the surface energy compared to those by Nuriel et al. [54]. According to these calculations, in unmodified blends, the CNTs stay in PC when adding 0.2 wt.% RC. After adding 2 or 20 wt.% the CNTs are expected to localize in SAN. The calculations using CNT surface energy values of Nuriel et al. [54] result in the opposite behavior. However, the changes in the behavior of the different nanotubes used in our study cannot be reflected by such calculations. In summary, the calculations and theoretical considerations provide only a limited indication of whether the localization change upon addition of RC to SAN is induced by altered surface energy values, but there is a hint in this direction.

Therefore, the proposed main effect is a strong interaction and reactive coupling of the functional surface groups of the CNTs with the MA groups inside the SAN-RC. When mixing all components together, SAN (or SAN-RC) softens first enabling the CNTs to meet MA groups and to interact with them. Even if they want to migrate towards PC, they are somehow fixed in the SAN-RC component and cannot migrate. A strong indication for that is given in our previous paper [47], where a blend containing 2 wt.% RC and 5 wt.% CNTs with localization of NC3152 (NH_2_ functionalized) in both components was diluted to get a blend with 0.5 wt.% and 0.2 wt.% RC in which this localization remained. However, when producing the diluted blend directly, localization in PC was observed. This illustrates an irreversible coupling, such as adsorption or covalent bonding of the RC on the CNT surface, which is also supported by the high electrical resistivities as discussed previously [47].

### 3.2. Differences Between the Behavior of Different CNTs

Interestingly, differences in the localization are seen when comparing the behavior of the different studied CNTs. While, in the first study, NH_2_-functionalized MWCNTs were used to induce the expected reaction with MA groups of SAN-RC, it was surprising that the corresponding unfunctionalized MWCNTs and other MWCNT types behave in the same manner.

In order to clarify the reason for that, IR, Raman and XPS studies of the CNTs were done to see the structural differences as well as differences in the functionality. It is expected that the surface functional groups and the defect density may play a role on the localization behavior.

The comparison of the IR spectra of unfunctionalized MWCNT NC3150 and functionalized MWCNT NC3152 shown in Figure S4 evidenced carboxylic groups seen at the peak at 1734 cm^−1^ in NC3150. The band assignment is shown in Table S3. This result indicates that functional groups exist in the chemical vapor deposition (CVD) produced nominally non-functionalized MWCNTs, which is in accordance with reports in literature [55]. It may be assumed that the reason for these bands lies in the defects and functional groups existent on the surface of the CVD grown MWCNTs even after the purification step involved in the production of the NC3150 series. Such oxygen based functional groups in MWCNTs were reported to be typical for products produced by the CVD method [56,57]. Therefore, the results about surface functionalities that can react with MA of SAN-RC can explain the selective localization of both MWCNT types in SAN-RC.

**Figure 8:**
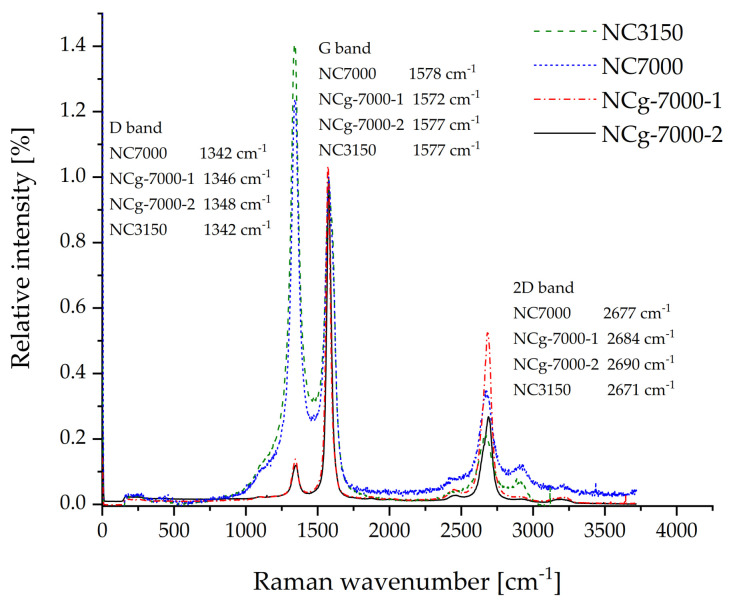
Raman spectra of NC3150, NC7000, NCg-7000-1, and NCg-7000-2, all normalized to the G-band.

Raman spectroscopy uses the D- and G-bands for the evaluation of order/disorder and defects. The higher the D/G intensity ratio, the greater the disorder in MWCNTs and the more defects in SWCNTs. The Raman spectra of the Nanocyl™ MWCNTs, as shown in Figure 8, show a much higher D than G band for the unfunctionalized NC3150 and NC7000, which reversed after the graphitization step. This indicates that disordered carbon and surface groups were removed by this step. A more perfect CNT structure and a lower defect density of the graphitized MWCNTs are achieved; however, the graphitized NC7000 also are not completely defect free as was intended. The intensity ratio between both bands, I_D_/I_G_, reduces from 1.28 for NC7000 and 1.42 for NC3150 to 0.11 for both NCg-7000. Thereby, no significant difference between the two graphitization steps of NC7000 can be observed.

**Figure 9:**
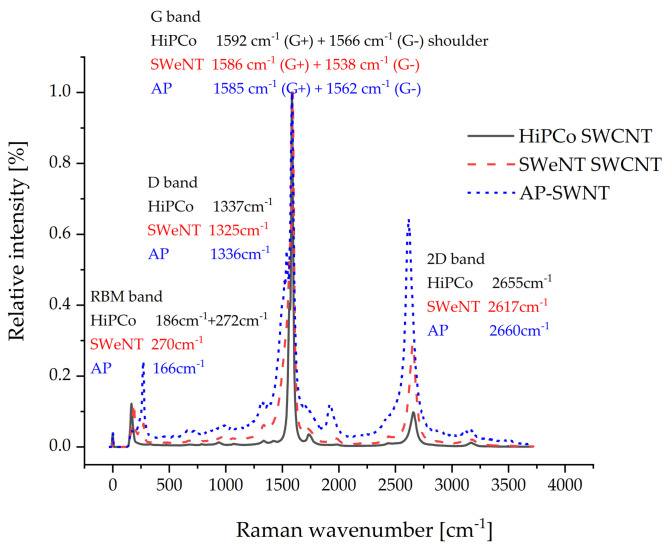
Raman spectra of HiPCo^TM^, SWeNT, and AP-SWNTs, all normalized to the G-band.

The Raman spectra of the non-functionalized SWCNTs are shown in Figure 9 and illustrate that the compared SWCNTs differ in their structure. By inspection of the different radial breathing mode bands, it is seen that the CNTs were produced by different synthesis methods. All SWCNTs show the typical two-peak G band, designated as G^+^ and G^−^. For AP-SWNT the D band is missing, indicating (along with the pronounced G band with low half width) the typical sp^2^ hybridization of carbon without defects. The I_D_/I_G_ ratio for HiPCo^TM^-SWCNT is 0.04 and for SWeNT-SWCNT 0.07 indicating some defect structures in the form of sp^3^ hybridized carbon on the surface of the SWCNTs.

**Figure 10:**
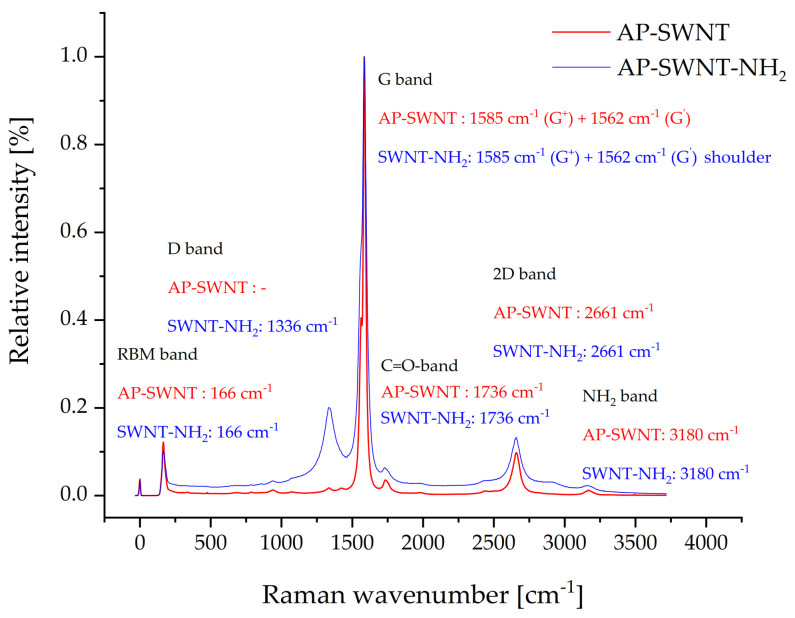
Comparison of Raman spectra of AP-SWNT and AP-NH_2_-SWNT materials, both normalized to the G-band.

The comparison of the NH_2_-functionalized AP-SWNTs with their parent SWCNT, as shown in Figure 10, indicates the appearance of a clear D band at 1336 cm^−1^ which can be related to the oxidation and amino-functionalization. The I_D_/I_G_ ratio is here 0.18 and confirms that. Both spectra show bands at 1736 cm^−1^ and 3180 cm^−1^ indicating the presence of carboxyl and amino groups on the surface of both AP-SWNT types.

In order to quantify the oxygen and other elements’ contents indicating functional groups in the used CNT materials, XPS analysis was performed. A summary of the most important ratios, namely [O]:[C]│_spec_, [N]:[C]│_spec_ and [O]:[C]│_org_ (indicating the amount of oxygen which is covalently bonded to carbon) is given in Table 2. Selected XPS spectra are given in Figure S5. SWCNTs naturally have a higher surface area and thus a higher number of functionalities per carbon atom and also bear the elements of the catalysts of the synthesis process or the agents used for removal of the catalyst particles and purification. Due to the remaining catalyst, the specific oxygen/carbon ratio [O]:[C]│_spec_ was not used for comparison, but rather the organic oxygen/carbon ratio [O]:[C]│_org_.

Comparing the [O]:[C]│_spec_ of the Nanocyl^TM^ products, the oxygen content is relatively high and increases during the purification and shortening step from NC7000 to NC3150. In NC3152, the sum of the elemental concentration of oxygen and nitrogen is the same as that for NC3150. Thus, the high number of functional groups, able to react with MA of SAN-RC, can explain the localization behavior similarity of all three MWCNT types. The graphitization of NC7000 results in a significantly reduced ratio of oxygen to carbon [O]:[C]│_spec_ of 0.002 after the second graphitization step in contrast to 0.008 before graphitization. However, a complete removal did not occur and still functional groups are available which can interact with MA groups of RC provided the concentration of RC is high enough (≥2 wt.%). The MWCNTs of the type Baytubes^®^ show a slightly lower oxygen content compared to the Nanocyl MWCNTs, though higher than the graphitized types. Thus, they behave in a similar manner to the other MWCNT types.

Comparing the SWCNT types, the lowest ratio [O]:[C]│_org_ could be observed for HiPCo^TM^, followed by SWeNT^®^ and AP-SWNT materials. Whereas the AP-SWNT also contain nitrogen, its content is significantly higher in the amino functionalized derivate. The lowest content of functional groups (oxygen and nitrogen) of HiPCo^TM^ SWCNT delivers a suitable explanation as to why this CNT type could be selectively localized in the PC component in PC_60_/SAN_38_-RC_2_ blends with 0.5 wt.% CNTs, whereas at this CNT concentration, all other SWCNT types resulted in in partial localization in PC and SAN-RC. It may be assumed that the functionalization density of these SWCNTs is high enough to induce a reaction with the MA group of SAN-RC. As soon as an SWCNT is dispersed in SAN-RC and able to react with RC, a wrapping of RC chains around the single CNTs is expected which retains this CNT in SAN-RC.

In order to prove the possibility of the coupling of surface groups of the nanotubes with MA groups of the RC, samples of RC with 30 wt.% Nanocyl™ NC3150 were melt mixed, dissolved in chloroform and homogenized using ultrasound. The solid part was filtered and the washing in chloroform was repeated 10 times, until no remaining RC (indicated by a yellowing of the chloroform) could be observed anymore. This product was used to perform thermogravimetric analysis (TGA) up to 800 °C. The results are presented in Figure S6. Whereas the NC3150 did show only a very small mass loss (1.6 wt.%), the RC degrades nearly completely with a mass loss of 97.2 wt.%. The MWCNTs on which a coupling of RC was expected showed after heating to 800 °C a residual amount of 55.4 wt.% meaning that the mass loss of nearly 45 wt.% can be assigned to RC which is coupled irreversibly to the CNTs.

## 4. Materials and Methods

### 4.1. Materials

Polycarbonate Makrolon^®^ 2600 was obtained from Bayer MaterialScience AG, Leverkusen, Germany (now Covestro Deutschland AG) and poly(styrene-*co*-acrylonitrile) Luran^®^ 358N from BASF SE, Ludwigshafen, Germany (now provided by INEOS Styrolution Europe GmbH, Frankfurt, Germany). The main properties of interest for this study are summarized in Table 3.

As reactive component (RC) *N-*phenylmaleimide styrene maleic anhydride copolymer with the description DENKA IP MS-L2A (Denki Kagaku Kōgyō KK, Tokyo, Japan) was used. Figure S7 and Table S4 show the IR spectra and its evaluation of this material. DSC studies confirmed the full miscibility of RC with SAN, as shown in Figures S8 and S9, and Table S5, in the Supplementary Material.

In the blends, different multi-walled carbon nanotubes of Nanocyl™ (Nanocyl S.A., Sambreville, Belgium) and Baytubes^®^ (Bayer MaterialScience, Leverkusen, Germany), as well as single-walled carbon nanotubes HiPCo^TM^ (CNI, Houston, TX, USA), SWeNT^®^ CG100 (SWeNT, SouthWest NanoTechnologies Inc., Norman, Oklahoma USA), AP-SWNT (Carbon Solutions, Inc., Riverside, CA, USA) and amino-functionalized AP-SWNT-NH_2_, which were functionalized by a partner from the Instituto de Carboquimica, CSIC, Dept. of Nanotechnology, Zaragoza Spain), were incorporated. A summary is given in Table 4, additional properties according to data sheets or references are summarized in Table S6.

### 4.2. Preparation and Compositions

All materials were dried under vacuum at 80 °C for 12 h. The blend components (PC, SAN), carbon nanotubes, and the reactive component were melt-mixed in a one step-procedure using a 15 cm^3^ microcompounder (DSM Xplore, Geleen, The Netherlands) operated at 260 °C, 100 rpm and a mixing time of 5 min. To produce the selectively filled blends, 0.5 wt.% of the different CNTs were incorporated into the polymer blend components in one mixing step. Previous investigations had shown the independence of the final nanotube localization in co-continuous PC_60_/SAN_40_-blends on the order of compounding (pre-compounding in one of the components or simultaneous mixing). For this, PC/SAN-RC blends were prepared in the ratios of 60/20-20 wt.%, 60/38-2 wt.% or 60/39.8-0.2 wt.%. In addition, on the example of Nanocyl™ NC3150 the tuning efficiencies of the RC was studied. For this, RC was added in a second step to PC_60_/SAN_40_-_x_/MWCNTs blends by varying the RC amount (x = 20 wt.%, 2 wt.%, 0.2 wt.%). For additional investigations, 0.5 wt.% of different SWCNTs were incorporated in blends containing 2 wt.% and 20 wt.% RC or without RC.

### 4.3. Morphological Characterization

The morphology of the blend systems was characterized via transmission electron microscopy (TEM). The TEM investigations were performed on ultra-thin sections of nominally 80 nm thickness cut at room temperature from the extruded strands by an ultramicrotome (Leica Microsystems GmbH, Wetzlar, Germany). TEM LIBRA 120 (Carl Zeiss SMT AG, Oberkochen, Germany) and LIBRA 200 MC (Carl Zeiss SMT AG, Oberkochen, Germany) operated at acceleration voltages of 120 kV and 200 kV, respectively, were used for imaging. Oxygen and nitrogen element maps were acquired by energy-filtered TEM (EF-TEM) using the standard 3-windows method.

### 4.4. Characterization of CNTs

All XPS studies were carried out by means of an Axis Ultra photoelectron spectrometer (Kratos Analytical, Manchester, UK). The spectrometer was equipped with a monochromatic Al Kα X-ray source of 300 W at 15 kV. The kinetic energy of photoelectrons was determined with hemispheric analyzer set to pass energy of 160 eV for wide-scan spectra and 20 eV for high-resolution spectra. Employing Scotch double-sided adhesive tape (3M Company, Maplewood, MN, USA) the powdery samples were prepared as thick films on a sample holder enabling the samples’ transport in the recipient of the XPS spectrometer. During all measurements, electrostatic charging of the sample was avoided by means of a low-energy electron source working in combination with a magnetic immersion lens. Later, all recorded peaks were shifted by the same value that was necessary to set the C 1s peak to 283.99 eV [70]. Quantitative elemental compositions were determined from peak areas using experimentally determined sensitivity factors and the spectrometer transmission function. Spectrum background was subtracted according to Shirley [71]. The high-resolution spectra were deconvoluted by means of the Kratos spectra deconvolution software. Free parameters of component peaks were their binding energy (BE), height, full width at half maximum, and the Gaussian–Lorentzian ratio.

The IR spectra of CNTs and RC were measured with the FTIR spectrometer Vertex 80v (Bruker Optics, Ettlingen, Germany) using the KBr disc method in the wavelength range from 400 cm^−1^ to 4000 cm^−1^ with a resolution of 2 cm^−1^ and 200 scans. All spectra were baseline corrected.

The SWCNTs and the Nanocyl™ MWCNTs were characterized by RAMAN spectroscopy using the RAMAN microscope alpha300R (WITec, Ulm, Germany) with 1 mW laser power, 1 s integration time, 200 accumulations, and a 20× objective.

### 4.5. Other Characterization Methods

Melt rheology on the blend component and selected SAN/RC mixtures was performed using the Advanced Rheometric Expansion System (ARES) rotational rheometer (Rheometric Scientific Inc., Piscataway, NJ, USA) under a nitrogen atmosphere. Compression molded plates with a thickness of 1 mm were placed between the parallel measuring plates with a diameter of 25 mm. The applied deformation amplitude was 1% and frequency sweeps were performed between 0.05 rad/s and 100 rad/s, followed by a second sweep from high to low frequencies, which was used for interpretation. The measurements were controlled by the RSI Orchestrator rheometer application software.

Modulated differential scanning calorimetry (MDSC) on the SAN/RC mixtures was done using a DSC Q 1000 (TA Instruments, New Castle, PA, USA) under nitrogen gas atmosphere in a heating-cooling-heating cycle with ± 2 K/min with an amplitude of ±0.31 K with a period of 40 s between −10 °C and 270 °C. The curves shown are related to the second heating. The glass transition temperature T_g_ was obtained using the half-step-method from the reversing heat flow curves of the second heating.

Thermogravimetric analysis (TGA) of the extracted CNT material was performed using a TGAQ5000 (TA Instruments, New Castle, PA, USA) in the temperature range between 30 °C and 800 °C at 10 K/min under a nitrogen atmosphere.

## 5. Conclusions

The investigations showed that the addition of a reactive component RC containing maleic anhydride groups can change the localization of all CVD grown MWCNTs from PC to SAN in melt-mixed co-continuous PC/SAN-RC blends. Thereby, a certain content of RC is required to achieve the selective localization in the SAN-RC component. Interestingly, the investigated SWCNTs indicated different localization behavior compared to MWCNTs. The HiPCo^TM^ SWCNTs localized selectively in PC, even after addition of RC, whereas the as-produced SWCNTs SWeNT^®^ and AP-SWNT localized in both components of the PC_60_/SAN_38_-RC_2_ blends. In contrast, the amino functionalized AP-SWNT localized in SAN-RC.

It seems that the surface functionality of the CNT material plays a crucial role in the localization behavior. The investigations (IR, XPS, Raman) of the different CNTs showed that all non-functionalized CNTs also have oxygen-based functional groups on their surface. These are able to react with maleic anhydride once the nanotube comes into contact with the SAN-RC mixture, which softens at lower temperatures than the PC component. This is considered to be the reason for the similar behavior between nominally non-functionalized and functionalized MWCNTs. Among the SWCNT materials, the HiPCo^TM^ material has the lowest oxygen content and I_D_/I_G_ ratio (lowest defect density), which seems to be the reason for its localization in the PC.

Exemplarily, TGA showed a significant amount of polymer coupled to MWCNTs dissolved from the blends, evidencing chemical coupling or strong interactions between CNTs and RC as the reason for their localization behavior in SAN-RC.

## Figures and Tables

**Table 1:** Assignment of which blend component the various CNTs are localized in when 0.5 wt.% CNTs are added in a one-step melt blending process.

CNT Type	PC_60_/SAN_20_-RC_20_	PC_60_/SAN_38_-RC_2_	PC_60_/SAN_39.8_-RC_0.2_	PC_60_/SAN_40_
NC3152 [47]	SAN-RC	SAN-RC	PC	PC
NC3150	SAN-RC	SAN-RC	PC	PC *
NC7000	SAN-RC	SAN-RC	PC	PC [50]
NCg-7000-1	SAN-RC*	SAN-RC	n. s.	PC
NCg-7000-2	SAN-RC	SAN-RC*	n. s.	PC
Baytubes^®^ C150P	SAN-RC	SAN-RC	PC	PC *
Baytubes^®^ C150HP	SAN-RC	SAN-RC	PC	PC [20]
HiPCo™	n. s.	PC	n. s.	PC
SWeNT^®^ CG100	SAN-RC and PC	SAN-RC and PC	n. s.	PC
AP-SWNT	n. s.	SAN-RC and PC	n. s.	n. s.
AP-SWNT-NH_2_	n. s.	SAN-RC	n. s.	n. s.

n. s. not studied; * not shown here.

**Table 2:** XPS: Ratio of oxygen and nitrogen content of the CNTs.

CNT type	[O]:[C]│_spec_	[N]:[C]│_spec_	[O]:[C]│_org_^a^
NC3152	0.007	0.004	0.007
NC3150	0.011	–	0.011
NC7000	0.008	–	0.059
NCg-7000-1	0.011	Traces	0.011
NCg-7000-2	0.002	–	0.002
Baytubes^®^ C150P	0.003	–	0.002
Baytubes^®^ C150HP	0.007	–	–
HiPCo^TM^	0.024	Traces	0.018
SWeNT^®^ CG100	0.037	0.004	0.033
AP-SWNT	0.059	0.067	0.053
AP-SWNT-NH_2_	0.057	0.025	0.054

^a^ The part of oxygen bound to carbon ([O]: [C]|_org_) was estimated based on the assumption that all the metal oxides are present in the highest oxidation state of metal.

**Table 3:** Physical properties of the used polymers according to data sheets [58,59,60], melt viscosity measured by the authors (see Figure 7).

Property	Polycarbonate (PC)	Poly(styrene-*co*-acrylonitrile) (SAN)	DENKA IP MS-L2A (RC)
Density, 25 °C [g/cm^3^]	1.2	1.08	1.18
Complex melt viscosity η^*^ (at 100 rad/s, 260 °C) [Pa s]	1260	211	1818
Glass transition temperature T_g_ (DSC)[°C]	148	108	196

**Table 4:** List of the employed carbon nanotube types.

Name	Producer	Type	Functionality	Synthesis Process
NC3152 [61]	Nanocyl™, Sambreville, Belgium	MWCNT	NH_2_	CVD, purified, shortened
NC3150 [62]	Nanocyl™, Sambreville, Belgium	MWCNT	-	CVD, purified, shortened
NC7000 [63]	Nanocyl™, Sambreville, Belgium	MWCNT	as prepared	CVD, as produced
NCg-7000-1	Nanocyl™, Sambreville, Belgium	MWCNT	-	CVD, graphitized at IPF
NCg-7000-2	Nanocyl™, Sambreville, Belgium	MWCNT	-	CVD, graphitized at IPF
Baytubes^®^ C150P [64]	Bayer MaterialScience, Leverkusen, Germany	MWCNT	-	CVD
Baytubes^®^ C150HP [65]	Bayer MaterialScience, Leverkusen, Germany	MWCNT	-	CVD, high purity
HiPCo™	Carbon Nanotechnologies Inc (CNI), Houston, TX, USA	SWCNT	as prepared	CVD, Fe(CO)_5_ [66]
SWeNT^®^ CG100 [67]	SWeNT, SouthWest NanoTechnologies Inc., Norman, OK, USA	SWCNT	as prepared	CoMoCAT^®^ process, CVD fluidized bed
AP-SWNT [50,68]	Carbon Solution Inc., Riverside, CA, USA	SWCNT	as prepared	electric arc, Ni/Y catalyst
AP-SWNT-NH_2_	Carbon Solution Inc., Riverside, CA, USA modified by CSIC, Spain, acc. to [69]	SWCNT	NH_2_	electric arc, Ni/Y catalyst

## Data Availability

The data presented in this study are available on request from the corresponding author.

## Supplementary Materials

The following are available online, Figure S1: Localization of NC g-7000-2 in the PC_60_/SAN_20_-RC_20_ blend demonstrated by EF-TEM. Figure S2: Localization of AP-SWNT in the PC_60_/SAN_38_-RC_2_ blend demonstrated by EF-TEM. Figure S3: Localization of AP-SWNT-NH_2_ in the PC_60_/SAN_38_-RC_2_ blend demonstrated by EF-TEM. Figure S4: IR spectra of MWCNTs Nanocyl™ NC3150 and Nanocyl™ NC3152. Figure S5: Wide-scan, C 1s and N 1s high-resolution XPS spectra, Figure S6: Study of the thermal degradation behavior (TGA) of RC, MWCNTs Nanocyl™ NC3150 and the reaction product between RC and NC3150, Figure S7: IR spectrum of *N*-phenylmaleimide styrene maleic anhydride copolymer, Figure S8: MDSC heating curves (second heating) of different SAN/RC mixtures. Figure S9: Glass transition temperature T_g_ for different SAN/RC mixtures, Table S1: Surface energy of the blend polymers and different SAN/RC mixtures with their polar (σ_p_) and disperse (σ_d_) parts, Table S2: Interfacial energies between the polymer blend partners, Table S3: Band assignment based on the IR spectra of MWCNTs Nanocyl™ NC3150 and Nanocyl™ NC3152, Table S4: Band assignment based on the IR spectra of *N*-phenylmaleimide styrene maleic anhydride, Table S5: Glass transition temperatures T_g_ and heat capacity Δcp of different SAN/RC mixtures, Table S6: Selected properties of the used nanotube materials according to their data sheets and additional references.

## Sample Availability

Samples of the composites are available from the authors.

## References

[1] Paul D.R., Newman S. (1978). Polymer Blends.

[2] Utracki L.A., Favis B.D. (1989). Polymer alloys and blends. Handbook of Polymer Science and Technology.

[3] Pötschke P., Paul D.R. (2003). Formation of Co-continuous structures in melt-mixed immiscible polymer blends. J. Macromol. Sci. Polym. Rev..

[4] Niebergall U., Bohse J., Schürmann B.L., Seidler S., Grellmann W. (1999). Relationship of fracture behavior and morphology in polyolefin blends. Polym. Eng. Sci..

[5] Willemse R.C., Speijer A., Langeraar A.E., Posthuma de Boer A. (1999). Tensile moduli of co-continuous polymer blends. Polymer.

[6] Veenstra H., Verkooijen P.C.J., van Lent B.J.J., van Dam J., de Boer A.P., Nijhof A.P.H.J. (2000). On the mechanical properties of co-continuous polymer blends: Experimental and modelling. Polymer.

[7] Kiran M.D., Govindaraju H.K., Jayaraju T., Kumar N. (2018). Review-effect of fillers on mechanical properties of polymer matrix composites. Mater. Today.

[8] Wolf C., Angellier-Coussy H., Gontard N., Doghieri F., Guillard V. (2018). How the shape of fillers affects the barrier properties of polymer/non-porous particles nanocomposites: A review. J. Membr. Sci..

[9] Breuer O., Sundararaj U. (2004). Big returns from small fibers: A review of polymer/carbon nanotube composites. Polym. Compos..

[10] Blackburn J.L., Ferguson A.J., Cho C., Grunlan J.C. (2018). Carbon-nanotube-based thermoelectric materials and devices. Adv. Mater..

[11] Popov V.N. (2004). Carbon nanotubes: Properties and application. Mater. Sci. Eng. R Rep..

[12] Nagy J.B., Coleman J.N., Fonseca A., Destrée A., Mekhalif Z., Moreau N., Vast L., Delhalle J. (2006). Carbon Nanotubes and Nanocomposites: Electrical, mechanical and flame-retardant aspects. Nanopages.

[13] Kausar A., Rafique I., Muhammad B. (2016). Review of applications of polymer/carbon nanotubes and epoxy/CNT composites. Polym. Plast. Technol. Eng..

[14] Sumita M., Sakata K., Asai S., Miyasaka K., Nakagawa H. (1991). Dispersion of fillers and the electrical conductivity of polymer blends filled with carbon black. Polym. Bull..

[15] Meincke O., Kaempfer D., Weickmann H., Friedrich C., Vathauer M., Warth H. (2004). Mechanical properties and electrical conductivity of carbon-nanotube filled polyamide-6 and its blends with acrylonitrile/butadiene/styrene. Polymer.

[16] Pötschke P., Bhattacharyya A.R., Janke A. (2003). Morphology and electrical resistivity of melt mixed blends of polyethylene and carbon nanotube filled polycarbonate. Polymer.

[17] Bose S., Bhattacharyya A., Kulkarni A., Pötschke P. (2009). Electrical, rheological and morphological studies in co-continuous blends of polyamide 6 and acrylonitrile-butadiene-styrene with multiwall carbon nanotubes prepared by melt blending. Compos. Sci. Technol..

[18] Wu D., Zhang Y., Zhang M., Yu W. (2009). Selective localization of multiwalled carbon nanotubes in poly(ε-caprolactone)/polylactide blend. Biomacromolecules.

[19] Otero-Navas I., Arjmand M., Sundararaj U. (2017). Carbon nanotube induced double percolation in polymer blends: Morphology, rheology and broadband dielectric properties. Polymer.

[20] Göldel A., Kasaliwal G., Pötschke P. (2009). Selective localization and migration of multiwalled carbon nanotubes in blends of polycarbonate and poly(styrene-acrylonitrile). Macromol. Rapid Commun..

[21] Wu D., Lin D., Zhang J., Zhou W., Zhang M., Zhang Y., Wang D., Lin B. (2011). Selective localization of nanofillers: Effect on morphology and crystallization of PLA/PCL blends. Macromol. Chem. Phys..

[22] Sun Y., Guo Z.-X., Yu J. (2010). Effect of ABS rubber content on the localization of MWCNTs in PC/ABS blends and electrical resistivity of the composites. Macromol. Mater. Eng..

[23] Zhang L., Wan C., Zhang Y. (2009). Investigation on the multiwalled carbon nanotubes reinforced polyamide 6/polypropylene composites. Polym. Eng. Sci..

[24] Wu M., Shaw L. (2004). On the improved properties of injection-molded, carbon nanotube-filled PET/PVDF blends. J. Power Sources.

[25] Pötschke P., Pegel S., Claes M., Bonduel D. (2008). A novel strategy to incorporate carbon nanotubes into thermoplastic matrices. Macromol. Rapid Commun..

[26] Baudouin A.-C., Devaux J., Bailly C. (2010). Localization of carbon nanotubes at the interface in blends of polyamide and ethylene-acrylate copolymer. Polymer.

[27] Cayla A., Campagne C., Rochery M., Devaux E. (2011). Electrical, rheological properties and morphologies of biphasic blends filled with carbon nanotubes in one of the two phases. Lancet.

[28] Shi Y., Li Y., Wu J., Huang T., Chen C., Peng Y., Wang Y. (2011). Toughening of poly(L-lactide)/multiwalled carbon nanotubes nanocomposite with ethylene-co-vinyl acetate. J. Polym. Sci. Part B.

[29] Lee C.J., Salehiyan R., Ham D.S., Cho S.K., Lee S.-J., Kim K.J., Yoo Y., Hyun K., Lee J.H., Choi W.J. (2016). Influence of carbon nanotubes localization and transfer on electrical conductivity in PA66/(PS/PPE)/CNTs nanocomposites. Polymer.

[30] Fenouillot F., Cassagnau P., Majesté J.-C. (2009). Uneven distribution of nanoparticles in immiscible fluids: Morphology development in polymer blends. Polymer.

[31] Salehiyan R., Ray S.S. (2019). Tuning the conductivity of nanocomposites through nanoparticle migration and interface crossing in immiscible polymer blends: A review on fundamental understanding. Macromol. Mater. Eng..

[32] Mamunya Y., Levchenko V., Boiteux G., Seytre G., Zanoaga M., Tanasa F., Lebedev E. (2016). Controlling morphology, electrical, and mechanical properties of polymer blends by heterogeneous distribution of carbon nanotubes. Polym. Compos..

[33] Xu L., Zhang B.-Y., Xiong Z.-Y., Guo Z.-X., Yu J. (2015). Preparation of conductive polyphenylene sulfide/polyamide 6/multiwalled carbon nanotube composites using the slow migration rate of multiwalled carbon nanotubes from polyphenylene sulfide to polyamide 6. J. Appl. Polym. Sci..

[34] Göldel A., Kasaliwal G.R., Pötschke P., Heinrich G. (2012). The kinetics of CNT transfer between immiscible blend phases during melt mixing. Polymer.

[35] Huang J., Mao C., Zhu Y., Jiang W., Yang X. (2014). Control of carbon nanotubes at the interface of a co-continuous immiscible polymer blend to fabricate conductive composites with ultralow percolation thresholds. Carbon.

[36] Huang Y., Ellingford C., Bowen C., McNally T., Wu D., Wan C. (2019). Tailoring the electrical and thermal conductivity of multi-component and multi-phase polymer composites. Int. Mater. Rev..

[37] Baudouin A.-C., Bailly C., Devaux J. (2010). Interface localization of carbon nanotubes in blends of two copolymers. Polym. Degrad. Stab..

[38] Zonder L., Ophir A., Kenig S., McCarthy S. (2011). The effect of carbon nanotubes on the rheology and electrical resistivity of polyamide 12/high density polyethylene blends. Polymer.

[39] Nishikawa R., Tamaki K., Notoya O., Yamaguchi M. (2020). Carbon nanotube localization at interface in cocontinuous blends of polyethylene and polycarbonate. J. Appl. Polym. Sci..

[40] Göldel A., Marmur A., Kasaliwal G.R., Pötschke P., Heinrich G. (2011). Shape-dependent localization of carbon nanotubes and carbon black in an immiscible polymer blend during melt mixing. Macromolecules.

[41] Pötschke P., Kretzschmar B., Janke A. (2007). Use of carbon nanotube filled polycarbonate in blends with montmorillonite filled polypropylene. Compos. Sci. Technol..

[42] Bai L., He S., Fruehwirth J.W., Stein A., Macosko C.W., Cheng X. (2017). Localizing graphene at the interface of continuous polymer blends: Morphology, rheology, and conductivity of continuous conductive polymer composites. J. Rheol..

[43] Bose S., Bhattacharyya A.R., Kodgire P.V., Misra A., Pötschke P. (2007). Rheology, morphology, and crystallization Behavior of melt-mixed blends of polyamide6 and acrylonitrile-butadiene-styrene: Influence of reactive compatibilizer premixed with multiwall carbon nanotubes. J. Appl. Polym. Sci..

[44] Prashantha K., Soulestin J., Lacrampe M.F., Krawczak P., Dupin G., Claes M. (2009). Masterbatch-based multi-walled carbon nanotube filled polypropylene nanocomposites: Assessment of rheological and mechanical properties. Compos. Sci. Technol..

[45] Lee G.-W., Jagannathan S., Chae H.G., Minus M.L., Kumar S. (2008). Carbon nanotube dispersion and exfoliation in polypropylene and structure and properties of the resulting composites. Polymer.

[46] Wang G., Qu Z., Liu L., Shi Q., Guo J. (2008). Study of SMA graft modified MWNT/PVC composite materials. Mater. Sci. Eng..

[47] Gültner M., Göldel A., Pötschke P. (2011). Tuning the localization of functionalized MWCNTs in SAN/PC blends by a reactive component. Compos. Sci. Technol..

[48] Zeng Y., Liu P., Du J., Zhao L., Ajayan P.M., Cheng H.-M. (2010). Increasing the electrical conductivity of carbon nanotube/polymer composites by using weak nanotube/polymer interactions. Carbon.

[49] Itkis M.E., Perea D.E., Niyogi S., Rickard S.M., Hamon M.A., Hu H., Zhao B., Haddon R.C. (2003). Purity evaluation of as-prepared single-walled carbon nanotube soot by use of solution-phase near-IR spectroscopy. Nano Lett..

[50] Liebscher M., Tzounis L., Pötschke P., Heinrich G. (2013). Influence of the viscosity ratio in PC/SAN blends filled with MWCNTs on the morphological, electrical, and melt rheological properties. Polymer.

[51] Feng J.Y., Chan C.M., Li J.X. (2003). A method to control the dispersion of carbon black in an immiscible polymer blend. Polym. Eng. Sci..

[52] Flaris V., Fletcher C., Gültner M., Pötschke P. Surface energy effects of PC/SAN/MWCNT blends with the addition of a reactive component. Proceedings of the 70th Annual Technical Conference Exhibition Conference Proceedings.

[53] Barber A.H., Cohen S.R., Wagner H.D. (2004). Static and dynamic wetting measurements of single carbon nanotubes. Phys. Rev. Lett..

[54] Nuriel S., Liu L., Barber A.H., Wagner H.D. (2005). Direct measurement of multiwall nanotube surface tension. Chem. Phys. Lett..

[55] Rausch J., Zhuang R.-C., Mäder E. (2010). Surfactant assisted dispersion of functionalized multi-walled carbon nanotubes in aqueous media. Compos. Part A.

[56] Datsyuk V., Kalyva M., Papagelis K., Parthenios J., Tasis D., Siokou A., Kallitsis I., Galiotis C. (2008). Chemical oxidation of multiwalled carbon nanotubes. Carbon.

[57] Lehman J.H., Terrones M., Mansfield E., Hurst K.E., Meunier V. (2011). Evaluating the characteristics of multiwall carbon nanotubes. Carbon.

[58] Covestro (Bayer), Makrolon 2600 PC Datasheet. http://materials.tecves.com/en/148/makrolon-2600.

[59] (2018). INEOS Styrolution Europe GmbH, CAMPUS® Datenblatt Luran® 358N—SAN. https://www.campusplastics.com/material/pdf/78486/Luran358N?sLg=en.

[60] Denki Kagaku Kōgyō K.K., Denka I.P. Maliimide Typi Heat Resistance Modifie Modifier. http://www.denka.co.kr/pdf/DENKA-IP_En.pdf.

[61] Nanocyl S.A. Technical Data Sheet: NC3152™, 25th January 2016, V01. http://www.nanocyl.com/wp-content/uploads/2016/02/Technical-Data-Sheet-NC3152-V01.pdf.

[62] Nanocyl S.A. Technical Data Sheet: NC3150™, 25th January 2016, V05. http://www.nanocyl.com/wp-content/uploads/2016/02/Technical-Data-Sheet-NC3150-V05.pdf.

[63] Nanocyl S.A. (2016). Technical Data Sheet: NC7000™, V08. https://www.nanocyl.com/wp-content/uploads/2016/07/DM-TI-02-TDS-NC7000-V08.pdf.

[64] Bayer Material Science (2009). Data Sheet Baytubes®C150P.

[65] Bayer Material Science (2007). Data Sheet Baytubes®C150HP.

[66] Bronikowski M.J., Willis P.A., Colbert D.T., Smith K.A., Smalley R.E. (2001). Gas-phase production of carbon single-walled nanotubes from carbon monoxide via the HiPco process: A parametric study. J. Vac. Sci. Technol. A.

[67] CoMoCAT® Single-wall Carbon Nanotubes. https://www.sigmaaldrich.com/technical-documents/articles/materials-science/nanomaterials/comocat-carbon-nanotubes.html.

[68] Product information AP-SWNT, Carbon Solutions Inc. https://www.carbonsolution.com/products/ap-swnt,.

[69] Lafuente E., Callejas M.A., Sainz R., Benito A.M., Maser W.K., Sanjuán M.L., Saurel D., de Teresa J.M., Martínez M.T. (2008). The influence of single-walled carbon nanotube functionalization on the electronic properties of their polyaniline composites. Carbon.

[70] Utech T., Pötschke P., Simon F., Janke A., Kettner H., Paiva M., Zimmerer C. (2020). Bio-inspired deposition of electrochemically exfoliated graphene layers for electrical resistance heating applications. Nano Express.

[71] Shirley D.A. (1972). High-Resolution X-Ray Photoemission spectrum of the valence bands of gold. Phys. Rev. B.

