# Supplementary material for: The Localization Behavior of Different CNTs in PC/SAN Blends Containing a Reactive Component
Source: Molecules. 2021 Mar 1;26(5):1312. doi: 10.3390/molecules26051312 (PMC7957507; doi:10.3390/molecules26051312)
Supplement: Supplementary file 1 [file molecules-26-01312-s001.pdf]

Supplementary materials to

The localization behavior of different CNTs in PC/SAN blends containing a reactive component

Marén Gültner[+], Regine Boldt, Petr Formanek, Dieter Fischer, Frank Simon, Petra Pötschke*

Leibniz-Institut für Polymerforschung Dresden e.V. (IPF Dresden), Hohe Str. 6, 01069 Dresden, Germany
* Correspondence: poe@ipfdd.de; Tel.: 0049 351 4658 395
+ present address: Sächsisches Textilforschungsinstitut e. V. (STFI), Annaberger Straße 240, 09125 Chemnitz, Germany

1. Energy-filtered (EF-TEM) investigations for the assignment of the blend components in the TEM images

Assignment of the blend components of PC_{60}/SAN_{38}-RC_2. In bright field image of TEM thin sections PC appears dark and smooth, SAN appears bright and ruffled area. This is confirmed by element mapping using EF-TEM. Since PC contains oxygen and does not contain nitrogen, and the SAN-RC contains substantially more nitrogen than oxygen, PC and SAN-RC can be discriminated by mapping oxygen and nitrogen distribution. In the nitrogen maps the bright areas are nitrogen rich (i.e. SAN-RC). In the oxygen maps the bright areas are oxygen rich (i.e. PC).

Bright Field Image | Nitrogen map | Oxygen map

PC_{60}/SAN_{20}-RC_{20}

NCg-7000-2

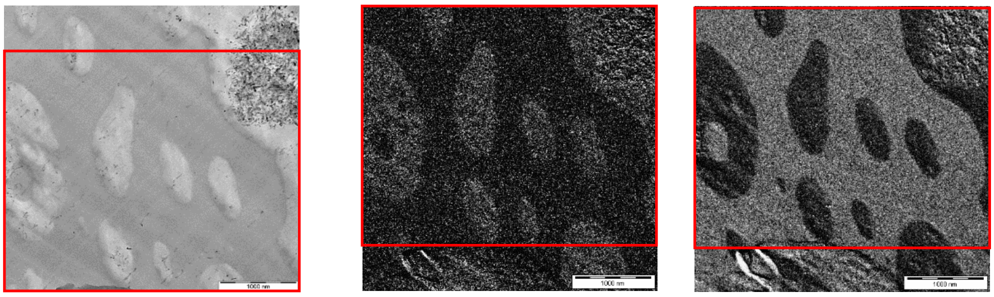

Figure S1. Localization of NCg-7000-2 in the PC_{60}/SAN_{20}-RC_{20} blend demonstrated by EF-TEM. NCg-7000-2 are localized in SAN-RC. The specimen drifted slightly between acquisition of bright field image and N and O maps. The rectangle marks the common area.

Bright Field Image | Nitrogen map | Oxygen map

PC_{60}/SAN_{38}-RC_2

AP-SWNT

Position 1

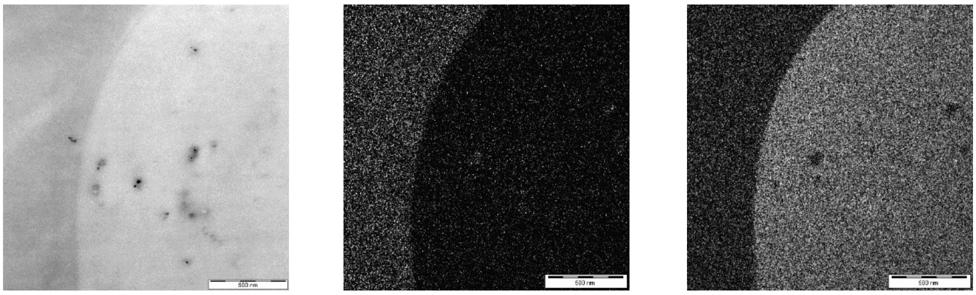

PC_{60}/SAN_{38}-RC_2

AP-SWNT

Position 2

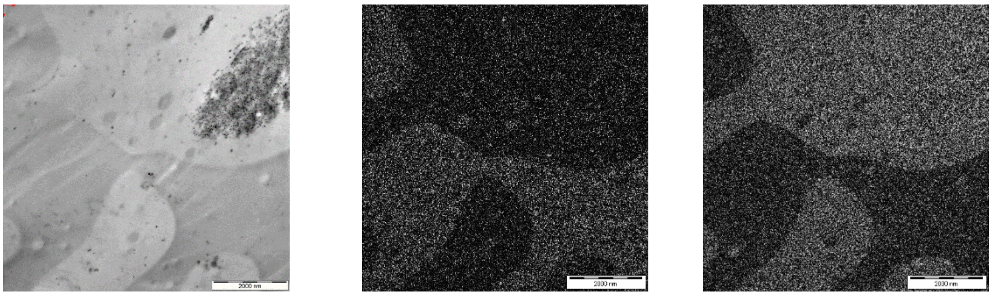

Figure S2. Localization of AP-SWNT in the PC_{60}/SAN_{38}-RC_2 blend demonstrated by EF-TEM. The main part of SWCNTs localized within PC, only single nanotubes stay in SAN-RC.

The assignment of the AP-NH_2-SWNTs or their agglomerates is much more complicated, since both the nitrogen of the amino groups and the oxygen caused by the oxidation during the functionalization of the CNTs or by the oxidized metal catalyst particles were detected in these areas. Isolated SWNTs could be directly assigned to the SAN-RC component (see also Figure 6d), whereas no isolated CNTs were found in PC **(Figure S3)**. In conclusion, it can be assumed that the individual AP-NH_2-SWNTs migrate to the SAN-RC component of the PC_{60}/SAN_{38}-RC_2 blend due to their functionalization during the melt-mixing process. In this context, it is also possible that the agglomerates of the AP-NH_2-SWNTs containing a large amount of catalyst particles localize between both blend components or in PC of the PC_{60}/SAN_{38}-RC_2 blends. A clear component assignment of those agglomerates is not possible due to their size and the contained oxygen and nitrogen elemental concentrations.

Bright Field Image | Nitrogen map | Oxygen map

PC_{60}/SAN_{38}-RC_2

AP-SWNT-NH_2

position 1

PC_{60}/SAN_{38}-RC_2

AP-SWNT-NH_2

position 2

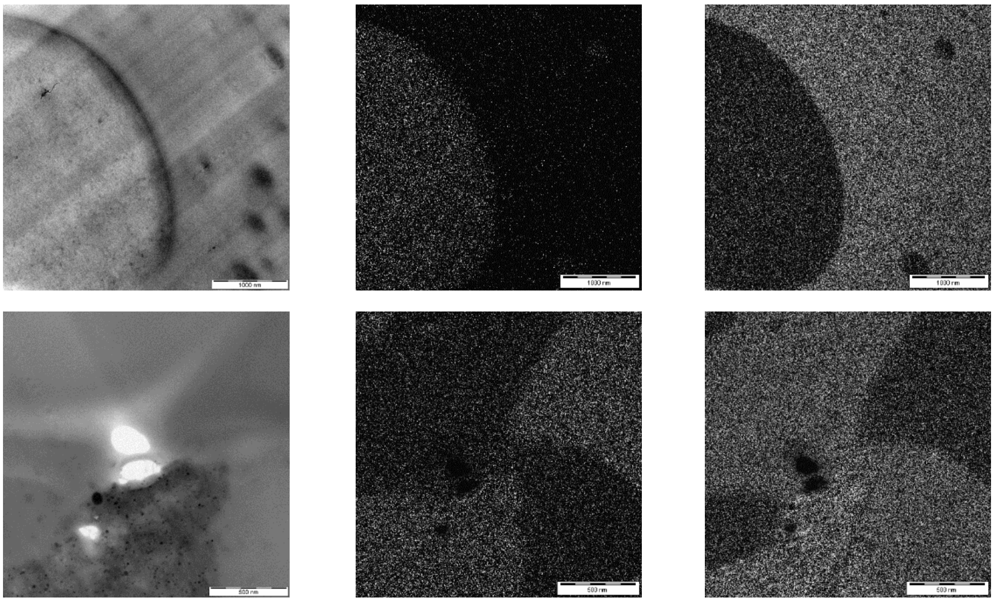

Figure S3. Localization of AP-SWNT-NH_2 in the PC_{60}/SAN_{38}-RC_2 blend demonstrated by EF-TEM. Individual amino-functionalized SWCNTs are localized within SAN-RC.

2. Calculation of surface energies and interfacial energies of RC modified PC/SAN/CNT blend composites

Table S1: Surface energy of the blend polymers and different SAN/RC mixtures with their polar (σ_P) and disperse (σ_d) parts as obtained from contact angle measurements of different liquids on compression molded polymer samples acc. to the methodology described in [1].

**Surface energy**	**SAN**	**PC**	**$SAN_{99.5}/RC_{0.5}$**	**SAN_{95}/RC_{5}**	**SAN_{50}/RC_{50}**
σ_P [mJ/m^2]	13.9	12.9	13.1	12. 6	11.2
σ_d [mJ/m^2]	20.9	21.0	20.8	20.9	18.6
σ [mJ/m^2]	34.8	33.9	33.9	33.4	29.8

Table S2: Interfacial energies between the polymer blend partners calculated based on values in Table S1 using the harmonic and geometric mean equations [2].

**Interfacial energy [mJ/m^2]**	**Acc. to harmonic mean equation**	**Acc. to geometric mean equation**
PC_{60}/SAN_{40}	0.042	0.021
$PC_{60}/SAN_{39.8}$-$RC_{0.2}$	0.003	0.002
PC_{60}/SAN_{38}-RC_{2}	0.004	0.002
PC_{60}/SAN_{20}-RC_{20}	0.263	0.132

3. IR spectra of MWCNTs Nanocyl™ NC3150 and Nanocyl™ NC3152 to proof the existence of functional groups on the surface of the nanotube materials

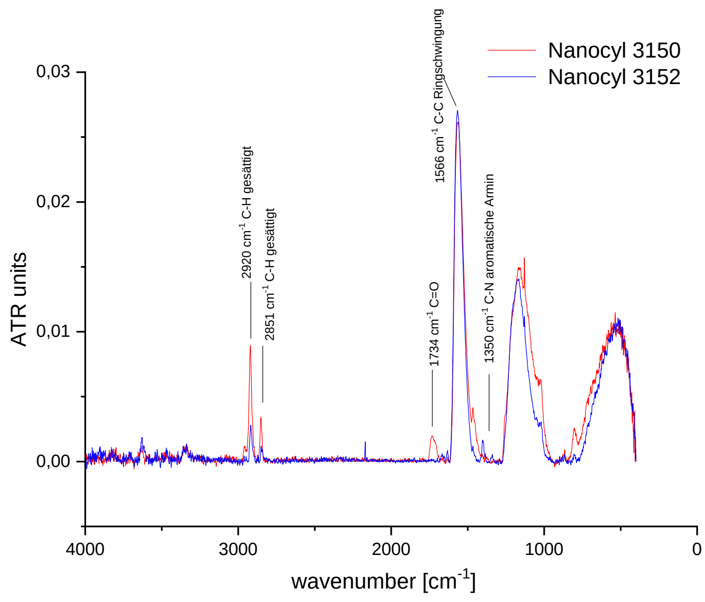

Figure S4: IR spectra of MWCNTs Nanocyl™ NC3150 and Nanocyl™ NC3152.

Table S3: Band assignment based on the IR spectra of MWCNTs Nanocyl™ NC3150 and Nanocyl™ NC3152.

**Wavenumber [cm-1]**	**Nanocyl™ NC3150**	**Nanocyl™ NC3152**
2920	CH_2/CH_3 stretching	CH_2/CH_3 stretching
2851	CH_2/CH_3 stretching	CH_2/CH_3 stretching
**1734**	**C=O**	-
1566	C=C ring	C=C ring
1493	C=C ring	
1350	-	C-N aromatic amine

4. Selected XPS spectra of the different CNT materials

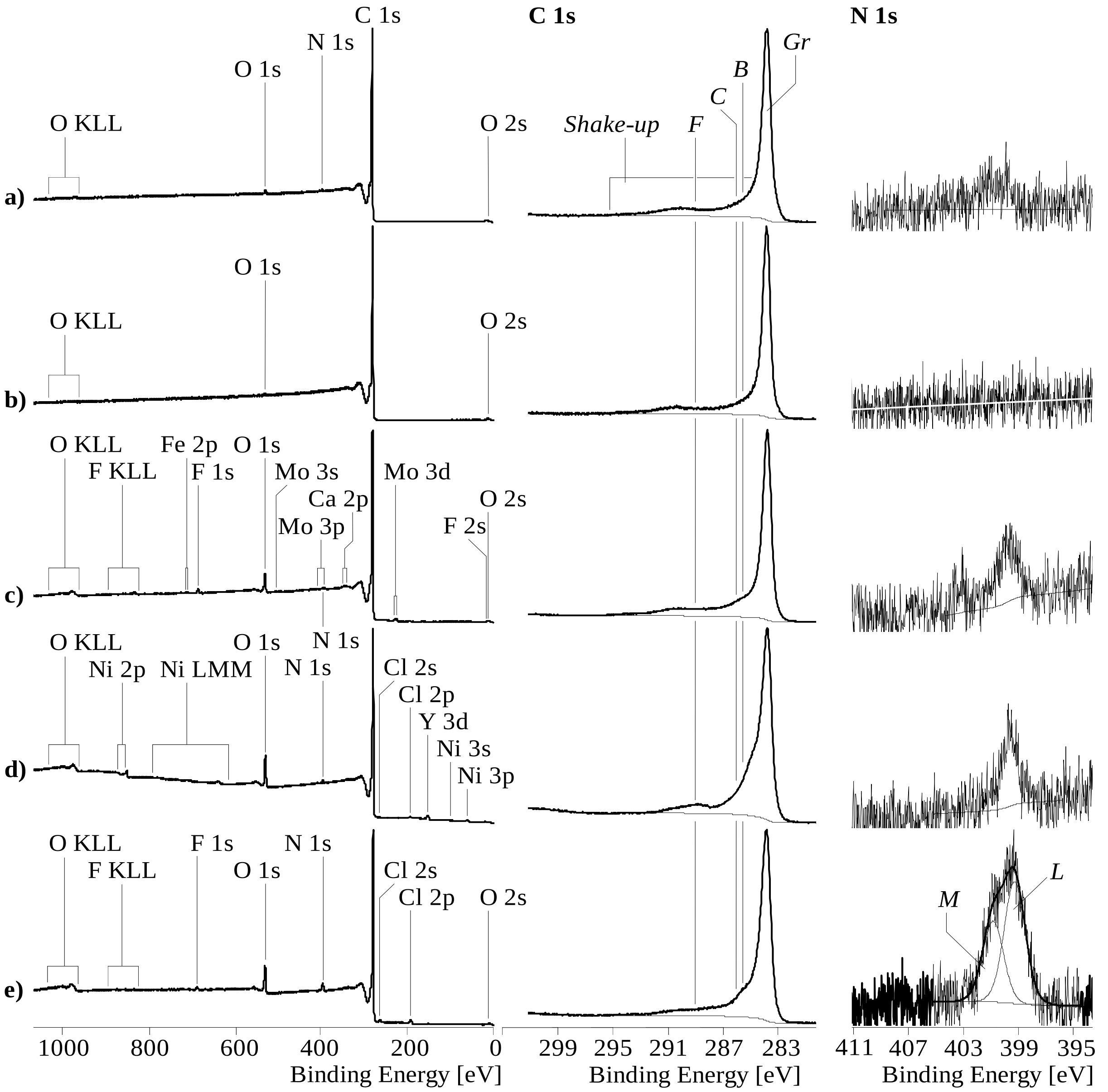

Figure S5. Wide-scan (first column), C 1s (second column) and N 1s (right column) high-resolution XPS spectra recorded from (a) NC3152, (b) Baytubes® C150P, (c) SWeNT® CG100, (d), AP-SWNT, and (e) AP-SWNT-NH_2. Italic letters in the high-resolution element spectra indicate the binding energy regions in which different functional groups are expected (more details are given in the text below).

In addition to the expected elements carbon and oxygen, the selected wide-scan spectra show the associated catalysts of the respective manufacturing process, which are still contained in the SWCNTs. They also give an impression of the amount of nitrogen, which was present on the surface of the different carbon nanotube species. It can be clearly seen that both the elements of the catalysts and the nitrogen – with the exception of the AP-SWNT-NH_2 sample – were only present as amounts of traces. Likewise, traces of the ions (F^-, $Cl^-$and Ca^{2+}) used to wash out the catalysts and purify the SWCNTs were found. In the HiPCo™ process (the spectra of the HiPCo ™ sample is not shown here), the common iron catalysts were washed out with hydrochloric acid (HCl). In the preparation of the SWeNT® SWCNTs via the CoMo-Cat® process, the Co/Mo catalyst was removed with the help of hydrogen fluoride (HF). The detection of molybdenum and iron in the SWeNT® CG100 sample shows that the purification process did not completely remove all of the residues of the catalysts. The HiPCo™ sample also contained traces of iron ($[Fe]:[C]|_{spec} \approx 0.003$) and molybdenum ($[Mo]:[C]|_{spec} \approx 0.003$).

All C 1s high-resolution spectra are characterized by strongly asymmetrical peaks. Steep rises on the lower-energy side are followed by pronounced tailings on the higher-energy side after the peak maximums, which were observed at 283.99 eV (*Gr* in **Figure S5**). The tailing result from high number of exited states in the graphite-like lattice of the carbon nanotubes. Photoelectrons escaped from the sp^2-hybridized carbon atoms in the electronic ground state forming the graphite-like lattices contributed to the main component that was labeled *Gr*. Each overlap of two p_z-orbitals forms a binding π-orbital and an anti-binding π*-orbital that are separated by an energy gap. Due to the geometric overlap of π-orbitals and π*-orbitals the linear combinations of the π-orbitals and π*-orbitals the gaps were converted in a quasi-continuum of electronic states that can be occupied with electrons (band structures). That means, each external energy can be used to transfer an electron from its ground state into an excited state, so that a large number of electronically excited states are permanently present in the graphite-like lattices of the carbon nanotubes. Photoelectrons escaped from carbon atoms in such excited states were collected as so-called *shake-up* peaks. The *shake-up* peaks in the C 1s spectra of the different carbon nanotube samples appear broad and fill almost the entire peak area at binding energies higher than 285.7 eV. Thus, component peaks resulting from functional groups are superimposed by the shake-up peaks. Nevertheless, from the shapes of the tailing C 1s peaks and the knowledge of the presence of carbon-bonded heteroatoms (detected in the wide-scan spectrum) it can be recognized that some of the carbon nanotube species carrying different functional groups. The region where the component peak resulting from amino groups ($\underline{C}$–N) was labelled with *B* (ca. 285.5 eV). Alcoholic and phenolic hydroxyl groups ($\underline{C}$–OH) were expected in the region marked with *C* (ca. 286.3 eV). In the C 1s spectra recorded from the SWeNT®-SWCNT samples, the presence of carboxylic acid groups (O=$\underline{C}$–OH) and their corresponding carboxylates (O=$\underline{C}$–O- ↔ -O–$\underline{C}$=O) can be clearly recognized by the missing saddle in *F* region (ca. 288.9 eV). Photoelectrons removed from saturated hydrocarbons were expected at 285.00 eV. Such from carbonyl carbon atoms of quinone-like ($\underline{C}$=O) and oxirane groups contribute to the C 1s spectra at ca. 288 eV. Obviously, their number seemed to be very small.

The N 1s high-resolution spectrum recorded from the AP-SWNT-NH_2 sample clearly showed two different binding states of nitrogen. From the binding energy values found for the two component peaks (*L* and *M*) it can be concluded that the nitrogen is organically bonded. Indeed, the binding energy value found for the component peak *L* (399.15 eV) seemed to be rather small for amino groups (C–$\underline{N}$). But it can be assumed that the nitrogen atoms of the majority of the amino groups providing their non-bonded electron pairs to the conjugated π-orbitals and participate from the high electron density there. Component peak *M* (400.89 eV) shows protonated amino groups (C–$\underline{N}^+$). The findings of the nitrogen's binding states can be transferred to the other N 1s spectra, which have much lower intensities. However, seeing the shapes of the N 1s spectra of the samples SWeNT® CG100 and AP-SWNT – specifically the regions around 403 eV – the presence of slightly oxidized nitrogen species cannot be excluded.

5. TGA study on RC coupled MWCNTs NC 3150

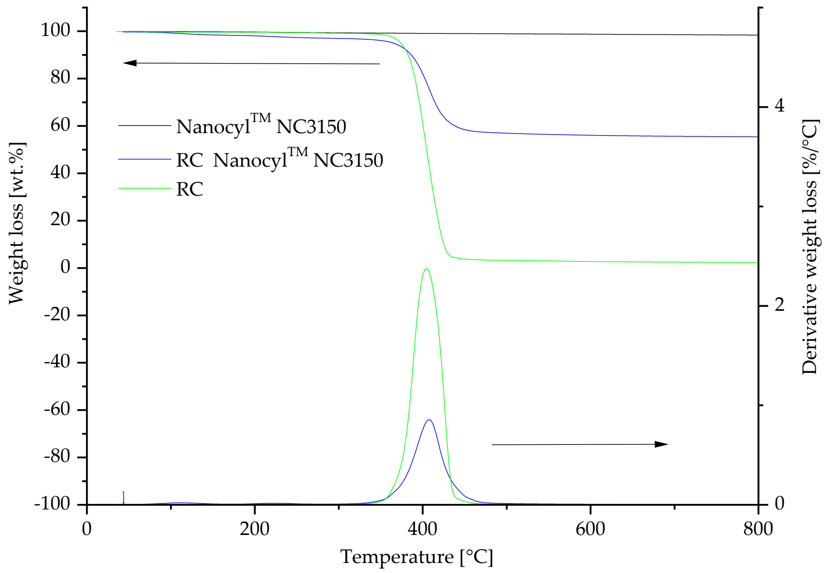

Figure S6. Study of the thermal degradation behavior (TGA) of RC, MWCNTs Nanocyl™ NC3150 and the reaction product between RC and NC3150 which could not be washed away on the CNT surface after ten washing cycles with chloroform.

6. IR spectrum and analysis of the reactive component (RC) to proof the maleic anhydride group

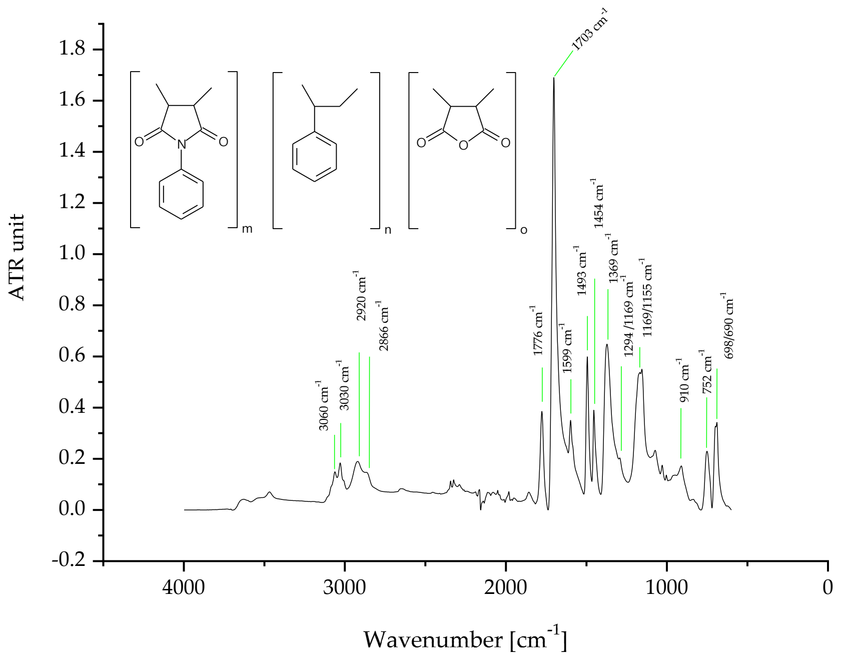

Figure S7. IR spectrum of *N*-phenylmaleimide styrene maleic anhydride copolymer.

Table S4: Band assignment based on the IR spectra of *N*-phenylmaleimide styrene maleic anhydride copolymer.

Wavenumber [cm^{-1}]	Vibration	Assignment
3060	CH=CH aromatic	N-Phenyl
3030	CH=CH aromatic	Styrene
2920	CH_2/CH_3 stretching	
2866	CH_2/CH_3 stretching	
1776	O=C-O-C=O resp. O=C-N-C=O	Diester
1703	C=O	Ketone
1599	C=C ring	
1493	C=C ring	
1454	CH_2/CH_3 bending	
1369	CH_3 bending	
1294/1169 (2 bands)	C-O-C stretching	Anhydride
1169/1155	C-N-C	*N*-phenylmaleimide
910	C-H deformation	Alkene
752	C-H deformation aromatic	
698/690	CH_2 bending	

7. MDSC behaviour of mixtures of SAN and RC to show their miscibility

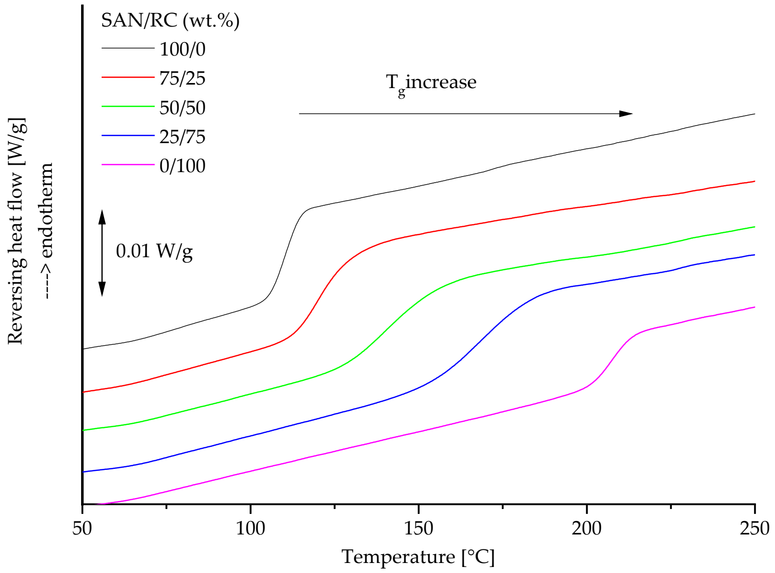

Figure S8. MDSC heating curves (second heating) of different SAN/RC mixtures.

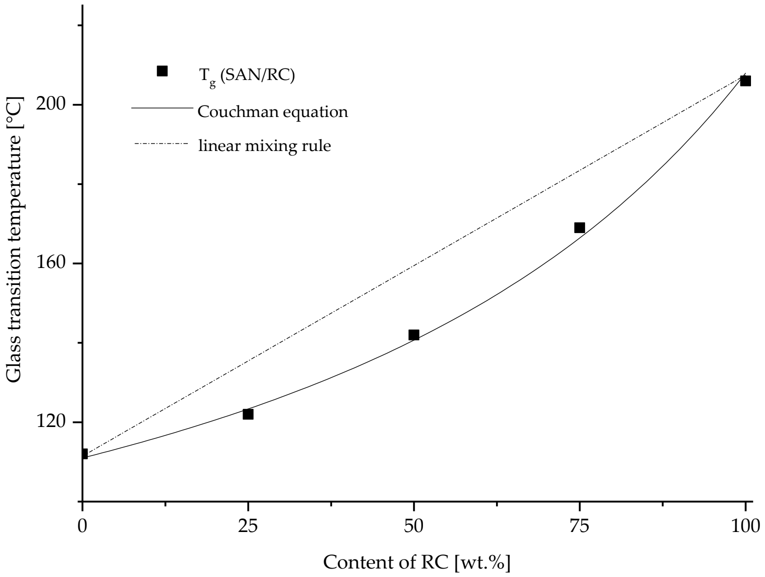

Figure S9. Glass transition temperature T_g for different SAN/RC mixtures.

Table S6.

Table S5: Glass transition temperatures T_g and heat capacity Δc_p of different SAN/RC mixtures.

Composition	T_g [°C]	Δc_p [J·g^{-1}·K^{-1}]
RC	208	
SAN/RC (25/75)	169	0.35
SAN/RC (50/50)	142	0.32
SAN/RC (75/25)	122	0.34
SAN	111	

As seen from **Figure S8**, only one glass transition exists for the blend systems, with the step in the normalized reverse heat flow (glass transition) being shifted to higher temperatures with increasing RC content. This indicates that the SAN/RC blends are miscible. To illustrate the relationship between glass transition temperature and blend concentration and to confirm miscibility, in **Figure S9** the glass transition temperatures in were plotted against the RC fraction.

The glass transition temperatures of the blends (**Table S6**) were compared with the general form of the Couchman equation [3] and the linear mixing rule. The prepared SAN/RC blends conform to Couchman's theoretical model. Thus, binary SAN/RC blends can be considered homogeneously miscible from a thermodynamic point of view.

8. Additional information about the used CNT materials

Table S6: Selected properties of the used nanotube materials according to their data sheets and additional references.

Name	Type	Mean diameter [nm]	Mean length [µm]	Carbon purity [%]	Other information
NC3152 [4]	MWCNT	9.5	<1.0	>95	NH_2 content 0.6%
NC3150 [5]	MWCNT	9.5	<1.0	>95	
NC7000 [6]	MWCNT	9.5	1.5	>80	
NCg-7000-1	MWCNT	9.5	1.5		
NCg-7000-2	MWCNT	9.5	1.5		
Baytubes® C150P [7-9]	MWCNT	5-20	1 - >10	>95	App. density 120-170 kg/m^3
Baytubes® C150HP [9,10]	MWCNT	5-20	1 - >10	>99	App. density 140-230 kg/m^3
HiPCo™ [1]	SWCNT	0.8-1.2	0.3-1.0	>95	
SWeNT® CG100 [11]	SWCNT	0.7-1.3		>70	Uniform chiral distribution
AP-SWNT [12]	SWCNT	1.4		60-70	
AP-SWNT-NH_2	SWCNT	1.4			Modified acc. to [13]

[1]Data sheet delivered with material in 2002, personal information CNI, June 2004

References:

1. Flaris, V.; Fletcher, C.; Gültner, M.; Pötschke, P. Surface Energy Effects of PC/SAN/MWCNT Blends with the Addition of a Reactive Component. *Antec 2012, Proceedings of the 70th Annual Technical Conference Exhibition Conference Proceedings, Orlando : Society of Plastic Engineers* **2012**.
2. Wu, S. *Polymer Interface and Adhesion*; Marcel Dekker Inc.: New York, 1982.
3. Couchman, P.R. Compositional Variation of Glass-Transition Temperatures. 2. Application of the Thermodynamic Theory to Compatible Polymer Blends. *Macromolecules* **1978**, *11*, 1156-1161, doi:10.1021/ma60066a018.
4. Nanocyl S.A., Technical Data Sheet: NC3152™, 25th January 2016, V01, http://www.nanocyl.com/wp-content/uploads/2016/02/Technical-Data-Sheet-NC3152-V01.pdf, assessed January 28th, 2021.
5. Nanocyl S.A. , Technical Data Sheet: NC3150™, 25th January 2016, V05, http://www.nanocyl.com/wp-content/uploads/2016/02/Technical-Data-Sheet-NC3150-V05.pdf, assessed January 28th, 2021.
6. Nanocyl S. A., Technical Data Sheet: NC7000™, V08, 12th July 2016, https://www.nanocyl.com/wp-content/uploads/2016/07/DM-TI-02-TDS-NC7000-V08.pdf, assessed January 28th, 2021.
7. Bayer MaterialScience AG, Data sheet Baytubes®C150P, Edition 2009-09-24.
8. Tessonnier, J.-P.; Rosenthal, D.; Hansen, T.W.; Hess, C.; Schuster, M.E.; Blume, R.; Girgsdies, F.; Pfänder, N.; Timpe, O.; Su, D.S., et al. Analysis of the structure and chemical properties of some commercial carbon nanostructures. *Carbon* **2009**, *47*, 1779-1798.
9. White, C.M.; Banks, R.; Hamerton, I.; Watts, J.F. Characterisation of commercially CVD grown multi-walled carbon nanotubes for paint applications. *Progress in Organic Coatings* **2016**, *90*, 44-53, doi:https://doi.org/10.1016/j.porgcoat.2015.09.020.
10. Bayer MaterialScience AG, Data sheet Baytubes®C150HP, Edition 2007-05-14.
11. CoMoCAT® Single-wall Carbon Nanotubes, https://www.sigmaaldrich.com/technical-documents/articles/materials-science/nanomaterials/comocat-carbon-nanotubes.html, assessed January 30th, 2021.
12. Product information AP-SWNT, Carbon Solutions Inc, https://www.carbonsolution.com/products/ap-swnt, assessed February 1st, 2021.

13. Lafuente, E.; Callejas, M.A.; Sainz, R.; Benito, A.M.; Maser, W.K.; Sanjuán, M.L.; Saurel, D.; de Teresa, J.M.; Martínez, M.T. The influence of single-walled carbon nanotube functionalization on the electronic properties of their polyaniline composites. *Carbon* **2008**, *46*, 1909-1917, doi:https://doi.org/10.1016/j.carbon.2008.07.039.
